# Human induced pluripotent stem cell-derived mesenchymal stem cells promote healing via TNF-α-stimulated gene-6 in inflammatory bowel disease models

**DOI:** 10.1038/s41419-019-1957-7

**Published:** 2019-09-26

**Authors:** Hongsheng Yang, Rui Feng, Qingling Fu, Shu Xu, Xiuxue Hao, Yun Qiu, Ting Feng, Zhirong Zeng, Minhu Chen, Shenghong Zhang

**Affiliations:** 1 Department of Gastroenterology, The First Affiliated Hospital, Sun Yat-sen University, Guangzhou, Guangdong, China; 2 Department of Gastroenterology, The Sixth Affiliated Hospital, Sun Yat-sen University, Guangzhou, Guangdong, China; 3 Otorhinolaryngology Hospital, The First Affiliated Hospital, Sun Yat-sen University, Guangzhou, Guangdong, China

**Keywords:** Mesenchymal stem cells, Inflammatory bowel disease

## Abstract

Therapeutic applications of tissue-derived mesenchymal stem cells (MSCs) are hindered by their limited expansion ability and variation across donors. Human induced pluripotent stem cell (iPSC)-derived MSCs show greater expandability and therefore offer potential for use in tissue repair therapies. Here we explored the regenerative effects of iPSC-MSCs and the mechanisms by which iPSC-MSCs promote mucosal healing via tumor necrosis factor-α-stimulated gene 6 (TSG-6) in mouse models of inflammatory bowel disease (IBD). Human iPSCs were induced to differentiate into MSCs following a clinically compliant protocol. The iPSC-MSC treatment promoted mucosal healing in colitic mice, accompanied by increased epithelial cell proliferation, CD44-positive cells, and Lgr5-positive cells. TSG-6 knockdown in iPSC-MSCs or blocking of hyaluronan–CD44 interactions by PEP-1 abrogated the therapeutic effects of iPSC-MSCs, whereas use of recombinant TSG-6 showed therapeutic effects similar to those of iPSC-MSCs. A mouse or patient-derived organoid culture system was developed. Organoids co-cultured with iPSC-MSCs showed increased epithelial cell proliferation, CD44-positive cells, and Lgr5-positive cells, which was abolished by TSG-6 knockdown. TSG-6-induced promoting effects in organoids were dependent on Akt activation and abrogated by the anti-CD44 antibody or MK2206. In conclusion, iPSC-MSCs promoted epithelial cell proliferation to accelerate mucosal healing in a murine colitis model via TSG-6 through hyaluronan–CD44 interactions in an Akt-dependent manner, demonstrating a patient-specific “off-the-shelf” format for IBD treatment.

## Introduction

Inflammatory bowel diseases (IBD), including Crohn’s disease (CD) and ulcerative colitis, are chronic relapsing diseases characterized by persistent intestinal inflammation and mucosal injuries^1^. Mucosal healing associated with improved long-term clinical outcomes has become a primary goal for IBD therapy^2^. A lack of mucosal healing usually predicts complications, including bleeding, perforation, and the development of fistulas, which require hospitalization and surgery. Unfortunately, only 30–50% of IBD patients treated with conventional therapies, including corticosteroids, immunomodulators, and even biologic agents, show mucosal healing^3^. Furthermore, conventional therapies to treat IBD are based on immune suppression; therefore, new therapeutic strategies to promote mucosal regeneration are needed^4,5^.

Mesenchymal stem cells (MSCs) are multipotent stromal cells derived from connective tissues^6^. MSCs used in treatments show advantages over other approaches because they constitute a stem cell niche, support the growth of tissue-specific stem cells, and promote tissue regeneration^7–9^. In addition, exogenous MSCs have shown regenerative potential in animal models of colitis, as well as in a clinical trial to treat IBD, especially perianal CD, via systemic or local delivery methods^10–14^. Of note, MSCs are hypoimmunogenic, and the use of allogeneic MSCs is usually safe^15,16^. Despite their promising therapeutic effects, tissue-derived MSCs have several weaknesses, such as their limited proliferative potential, standardization difficulty, loss of differentiation capacity, and decreased therapeutic efficacy during expansion^17^. Previously, we generated single cell-derived MSCs from human induced pluripotent stem cells (iPSCs)^18–20^ and showed that MSCs could be produced from iPSCs and expanded with high efficiency. Furthermore, we demonstrated the regenerative potential of iPSC-MSCs compared to that of bone marrow-derived MSCs in a mouse model of hindlimb ischemia^18^. Because we observed repair of other tissues in response to iPSC-MSCs, we further evaluated the therapeutic effects of human iPSC-MSCs on mucosal healing in mouse models of IBD in the present study.

Tumor necrosis factor-α-stimulated gene 6 (TSG-6) is a 30-kDa glycoprotein that is synthesized by MSCs and fibroblasts in an inflammatory state^21^. MSCs produce far more TSG-6 in response to inflammatory mediators than fibroblasts^22^. Whether the promotion of mucosal healing by iPSC-MSCs is also dependent on TSG-6 should be investigated. The therapeutic effects of TSG-6 demonstrated in mouse models of myocardial infarction, corneal injury, skin wounds, and arthritis can be explained, at least in part, by the anti-inflammatory properties of TSG-6^22–25^. Recently, studies have shown that TSG-6 may also participate in the activation of epithelial stem cells^26–28^. However, the mechanism by which TSG-6 activates epithelial stem cells and, in turn, promotes ulcer repair in murine colitis remains unclear. In the current study, we found that TSG-6 released by iPSC-MSCs plays an essential role in determining the therapeutic effects of iPSC-MSCs on murine colitis by stimulating intestinal epithelial proliferation through interactions between CD44 and hyaluronic acid (HA). These findings provide a critical link to understand the molecular mechanism underlying the regenerative properties of iPSC-MSCs and may contribute to developing patient-specific IBD therapies aimed at mucosal healing.

## Results

### iPSC-MSCs promote mucosal healing via TSG-6 in murine colitis models

**Fig. 1:**
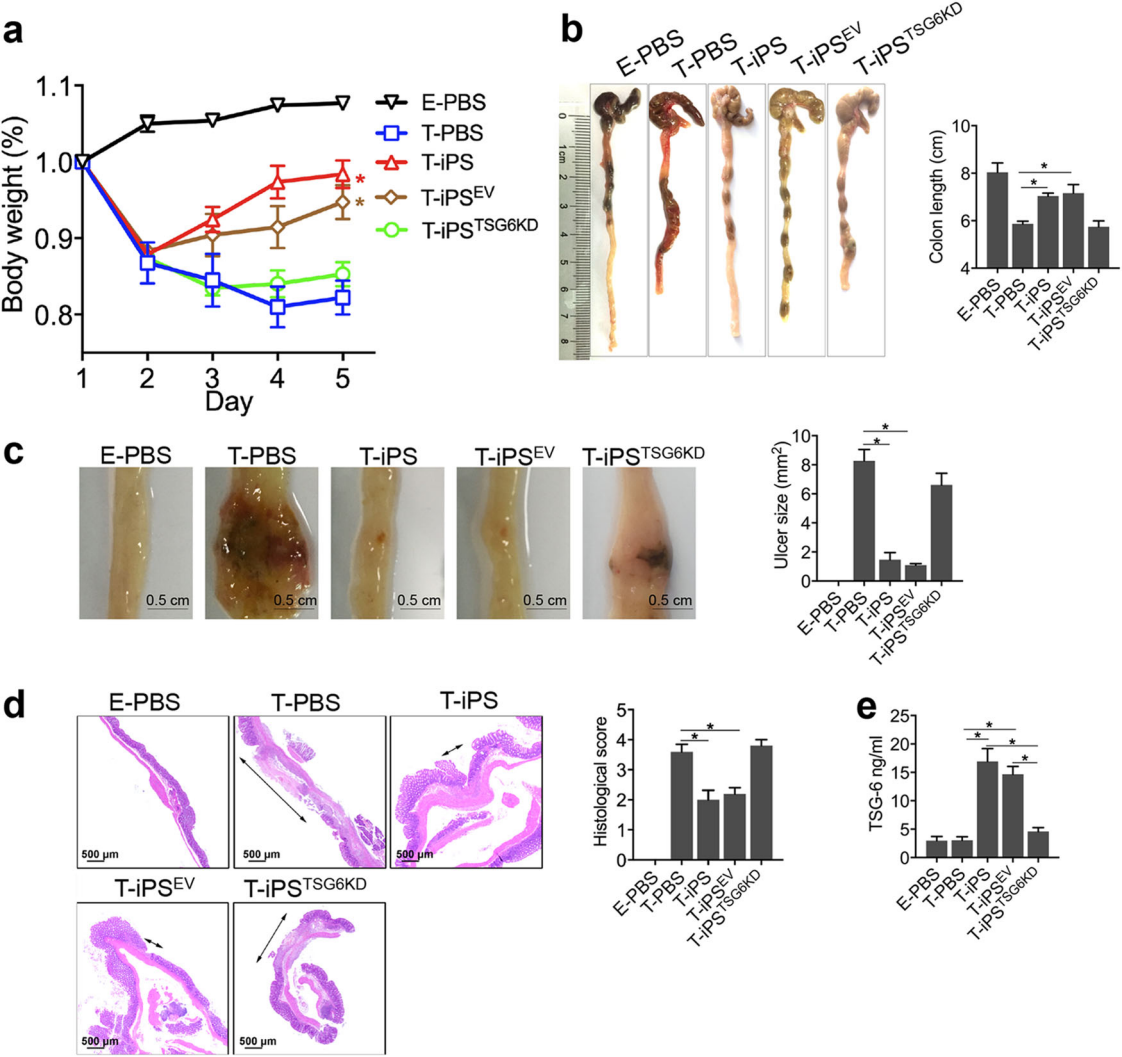
iPSC-MSCs promote mucosal healing via TSG-6 in murine colitis models. On the next day after intrarectal administration of TNBS, mice received intraperitoneal injections with 2 × 10^6^ iPSC-MSCs (T-iPS group), iPSC-MSCs^EV^ (T-iPS^EV^ group), iPSC-MSCs^TSG6KD^ (T-iPS^TSG6KD^ group), or PBS (T-PBS group), whereas healthy mice were given an injection of PBS as a negative control (E-PBS group). **a** Changes in body weight during the entire experiment showed as the percentage of the initial body weight at the start of the experiment. *P < 0.05 versus T-PBS group at day 5. **b** Gross morphology and length of colons in individual groups. **c** Representative photographs of gross colonic ulcers were shown. Colonic ulcer area was measured by the ImageJ software. **d** Representative histology images and histological scores in individual groups. Double arrowhead lines indicated colonic ulcers. **e** Serum TSG-6 protein levels in individual groups were determined by ELISA. *n* = 5 mice/group. *P < 0.05

To explore the therapeutic efficacy of iPSC-MSCs in colitis, a trinitrobenzene sulfonate (TNBS)-induced murine colitis model was developed. We found that a single intraperitoneal injection of iPSC-MSCs (2 × 10^6^ cells per mouse) significantly improved clinical parameters in colitic mice (T-iPS group) such as body weight and colon length when compared to colitic mice that were administered phosphate-buffered saline (PBS) (T-PBS group) (Fig. 1a, b). Notedly, ulcer sizes and histological scores improved significantly in the T-iPS group compared to those in the T-PBS group (Fig. 1c, d). There were no significant differences in clinical parameters and histological manifestations between control mice administered iPSC-MSCs and those given PBS (data not shown).

After intraperitoneal injection of luciferase-labeled iPSC-MSCs, in vivo imaging analyses revealed an intense signal in MSC-treated mice that was sustained through the remainder of the experiment (Supplementary Fig. 1a). Flow cytometry showed a high frequency of green fluorescent protein-labeled (GFP+) cells in the peritoneal cavities of mice and a relatively low frequency in colons (Supplementary Fig. 1b, c). No GFP+ cells were detected in the livers or spleens. Of note, more GFP+ cells appeared in the inflamed colons of MSC-treated colitic mice than in the colons of MSC-treated control mice. Furthermore, immunofluorescence assay confirmed the presence of GFP+ cells expressing TSG-6 in the colons of MSC-treated mice (Supplementary Fig. 1d).

We also noted that there were significantly elevated TSG-6 serum levels in MSC-treated colitic mice compared with control mice given PBS (Fig. 1e). Several studies have shown that TSG-6 accelerates tissue repair in many murine disease models, including models of corneal injury, myocardial infarction, and lung injury^22,24,29^. Therefore, to investigate whether iPSC-MSCs exerted therapeutic effects via TSG-6, we transfected iPSC-MSCs with a lentivirus containing short hairpin RNA (shRNA) targeting TSG-6 (Supplementary Fig. 2a–d) and evaluated the therapeutic effects of iPSC-MSCs on murine colitis after TSG-6 knockdown (iPSC-MSCs^TSG-6KD^). As expected, TSG-6 serum levels did not increase in colitic mice after iPSC-MSC^TSG-6KD^ injection (T-iPS^TSG-6KD^ group), indicating that iPSC-MSCs injected intraperitoneally into colitic mice were the source of increased serum TSG-6 (Fig. 1e). Moreover, iPSC-MSCs lost their therapeutic effects after TSG-6 knockdown (Fig. 1a–d).

To further validate the therapeutic effects of iPSC-MSCs, we also intraperitoneally injected a single dose of iPSC-MSCs (2 × 10^6^ cells per mouse) into an experimental murine colitis model induced by dextran sulfate sodium (DSS). Similar trends in symptom severity, ulcer size, and histological manifestations was observed in DSS-induced colitic mice treated with iPSC-MSCs (D-iPS group) compared to those treated with PBS (D-PBS group) (Supplementary Fig. 3a–e). In addition, iPSC-MSCs with TSG-6 knockdown did not show therapeutic effects in the DSS-induced colitis model.

**Fig. 2:**
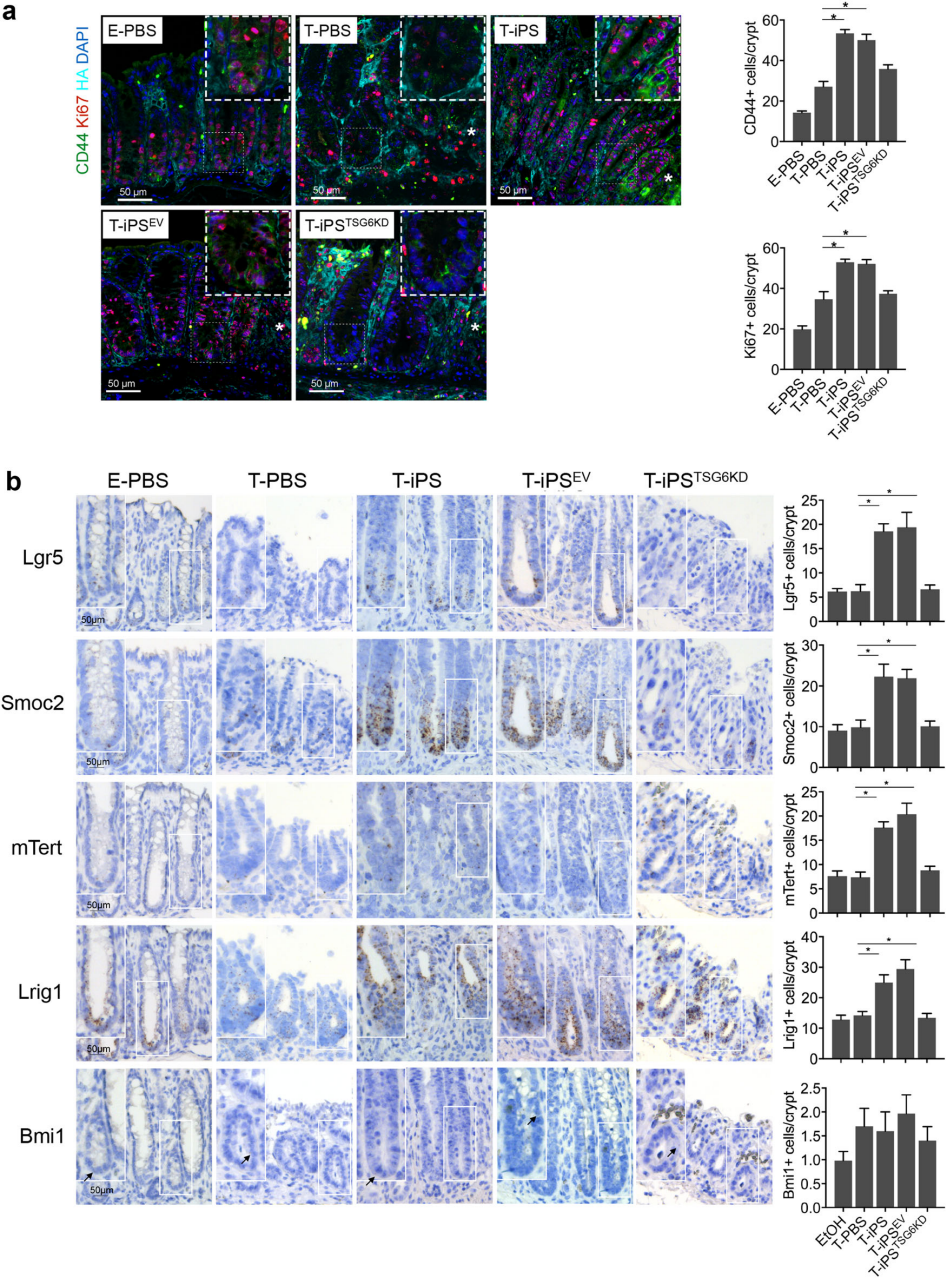
iPSC-MSCs promote epithelial cell proliferation via TSG-6 in murine colitis models. **a** Immunofluorescence staining for hyaluronic acid (HA), Ki67, and CD44. Asterisks indicated ulcer margins. The numbers of Ki67-positive cells or CD44-positive cells were counted from five glands at ulcer margins. **b** Serial sections in situ hybridized for cycling crypt base columnar cell markers (Lgr5, Smoc2) and quiescent +4 stem cell markers (mTert, Lrig1, and Bmi1). Bar graph shows the numbers of positively stained cells of the indicated markers per crypt in each group. Arrows indicated Bmi1-positive cells in the crypt. *n* = 5 mice/group. *P < 0.05

A previous study showed that TSG-6 modulates interactions between HA and cell surface receptor CD44^30^. CD44 is a recognized marker of intestinal epithelial stem cells^31^. Therefore, triple immunofluorescence staining was performed to evaluate intestinal epithelial stem cell proliferation and CD44–HA interactions after iPSC-MSC treatment. Immunofluorescence of the HA-binding protein showed that HA extended higher into colonic crypts during tissue repair (Fig. 2a, Supplementary Fig. 4). Importantly, the T-iPS group showed an increase in the number of CD44-positive (CD44+) epithelial cells and proliferating epithelial cells (Ki67-positive, Ki67+) per crypt at ulcer margins compared to that in the T-PBS group, whereas CD44+ epithelial cells and Ki67+ epithelial cells did not increase significantly in the T-iPS^TSG-6KD^ group (Fig. 2a). The difference in Ki67+ epithelial cell and CD44+ epithelial cell numbers was still significant even when inflammation levels were comparable between the T-PBS and T-iPS groups (Supplementary Fig. 5).

To further assess the impacts of iPSC-MSCs on the intestinal epithelial stem cell population, we performed RNA in situ hybridization (ISH) analyses. Intestinal epithelial stem cell populations comprise two cell types: cycling crypt base columnar (CBC) cells and quiescent +4 stem cells^32^. The RNA ISH analyses (Fig. 2b) showed that CBC stem cell markers (leucine-rich repeat-containing G-protein coupled receptor 5 [Lgr5], and SPARC-related modular calcium-binding protein 2 [Smoc2]) were enriched at the bottom of crypts, and the numbers of both Lgr5-positive (Lgr5+) cells and Smoc2-positive (Smoc2+) cells increased significantly in the T-iPS group compared with those in the T-PBS group. However, the patterns of expression for the +4 stem cell markers (mouse telomerase reverse transcriptase [mTert], leucine-rich repeats and immunoglobulin-like domains 1 [Lrig1], and B lymphoma Mo-MLV-insertion region 1 homolog [Bmi1]) varied. Bim1 and mTert were sporadically expressed throughout the crypt at very low levels, whereas expression of Lrig1 was enriched at the base of crypts and extended along the crypt axis. The numbers of both mTert-positive (mTert+) and Lrig1-positive (Lrig1+) cells increased significantly in the T-iPS group compared to those in the T-PBS group. In contrast, Bmi1-positive (Bmi1+) cells showed no significant differences between the T-iPS and T-PBS groups, and neither the CBC stem cell markers nor the +4 stem cell markers increased in the T-iPS^TSG-6KD^ group. In summary, these data suggested that iPSC-MSCs may accelerate crypt epithelial cell proliferation and activate CBC stem cells via TSG-6.

### iPSC-MSCs promote the proliferation of colonoids ex vivo via TSG-6

**Fig. 3:**
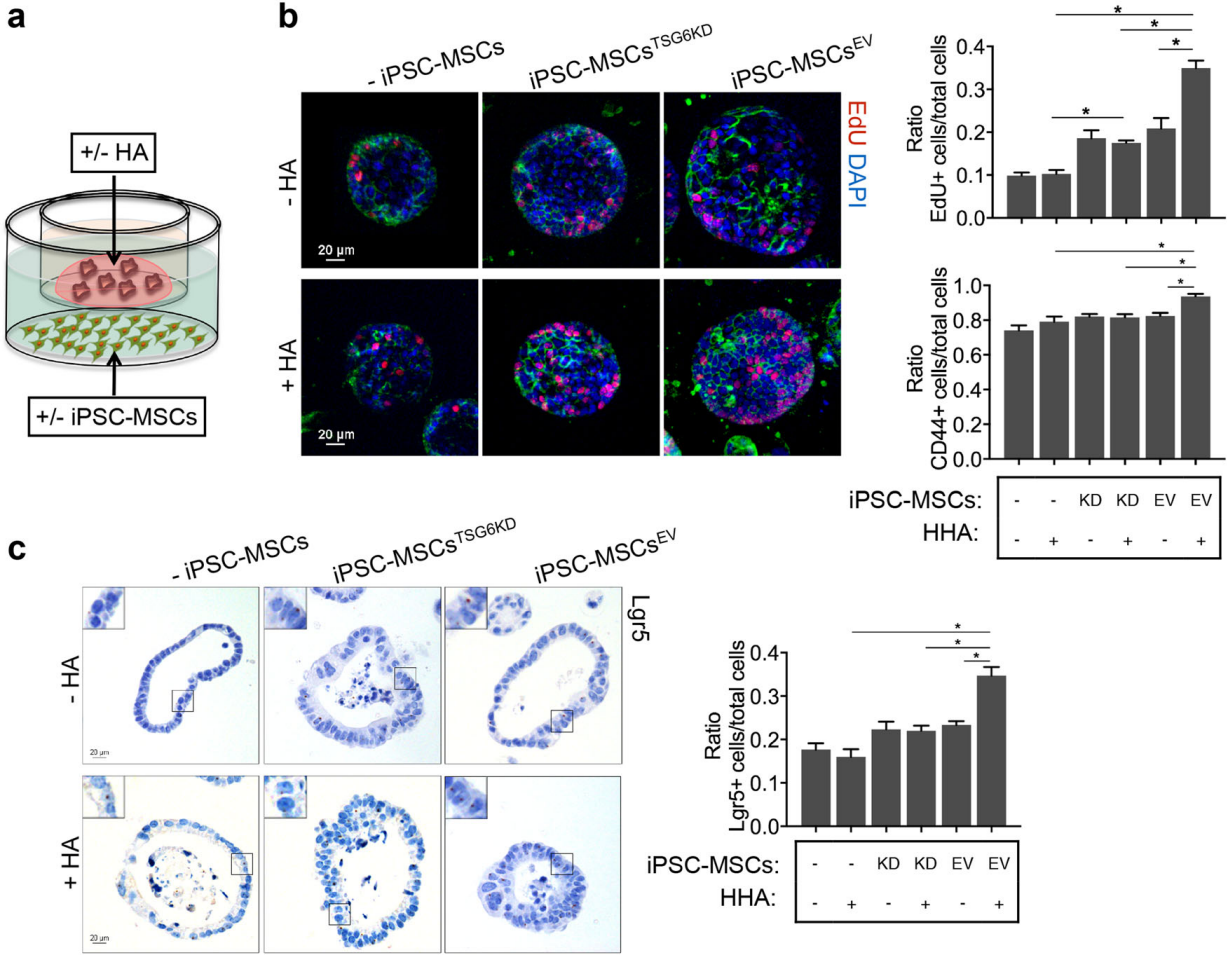
iPSC-MSCs promote the proliferation of colonoids ex vivo via TSG-6. **a** Schematic diagram of the coculture system with organoids and iPSC-MSCs. Murine colonoids plating on Transwell membranes were cocultured with iPSC-MSCs with or without TSG-6 knockdown (iPSC-MSC^EV^ or iPSC-MSCs^TSG6KD^). **b** Murine colonoids were immunostained with 5-ethynyl-20-deoxyuridine (EdU) (red), CD44 (green), and nucleus (blue). Total cell number, EdU-labeled nuclei, and CD44-positive cells were counted and results were expressed as EdU-positive cells/total cells ratio and CD44-positive cells/total cells ratio. **c** RNA in situ hybridization for the intestinal epithelial stem cell marker (Lgr5) in murine colonoids. Total cell number and Lgr5-positive cells were counted and results were expressed as Lgr5-positive cells/total cells ratio. *n* = 4 independent experiments. *P < 0.05

To confirm our observations in vivo, a coculture system of murine colonoids and iPSC-MSCs was used (Fig. 3a). The iPSC-MSCs were activated by preincubation with 100 ng/mL TNF-α for 16 h to increase the expression and secretion of TSG-6 before the coculture with colonoids (Supplementary Fig. 6a–c). Immunofluorescence staining of proliferative marker 5-ethynyl-20-deoxyuridine (EdU) revealed significantly induced proliferation of colonoids that were cocultured with activated iPSC-MSCs compared to those that were not cocultured with iPSC-MSCs (Fig. 3b). Furthermore, iPSC-MSCs also significantly increased the frequency of CD44+ cells and CBC stem cells (Lgr5+ cells) in colonoids (Fig. 3b, c). The iPSC-MSC-induced proliferation of colonoids was abrogated by knockdown of TSG-6 in iPSC-MSCs (Fig. 3b, c).

Collectively, the in vivo and the ex vivo experiments showed that iPSC-MSCs promoted crypt epithelial cell proliferation via TSG-6.

### TSG-6 and iPSC-MSCs promote mucosal healing in murine colitis through interactions between CD44 and HA

**Fig. 4:**
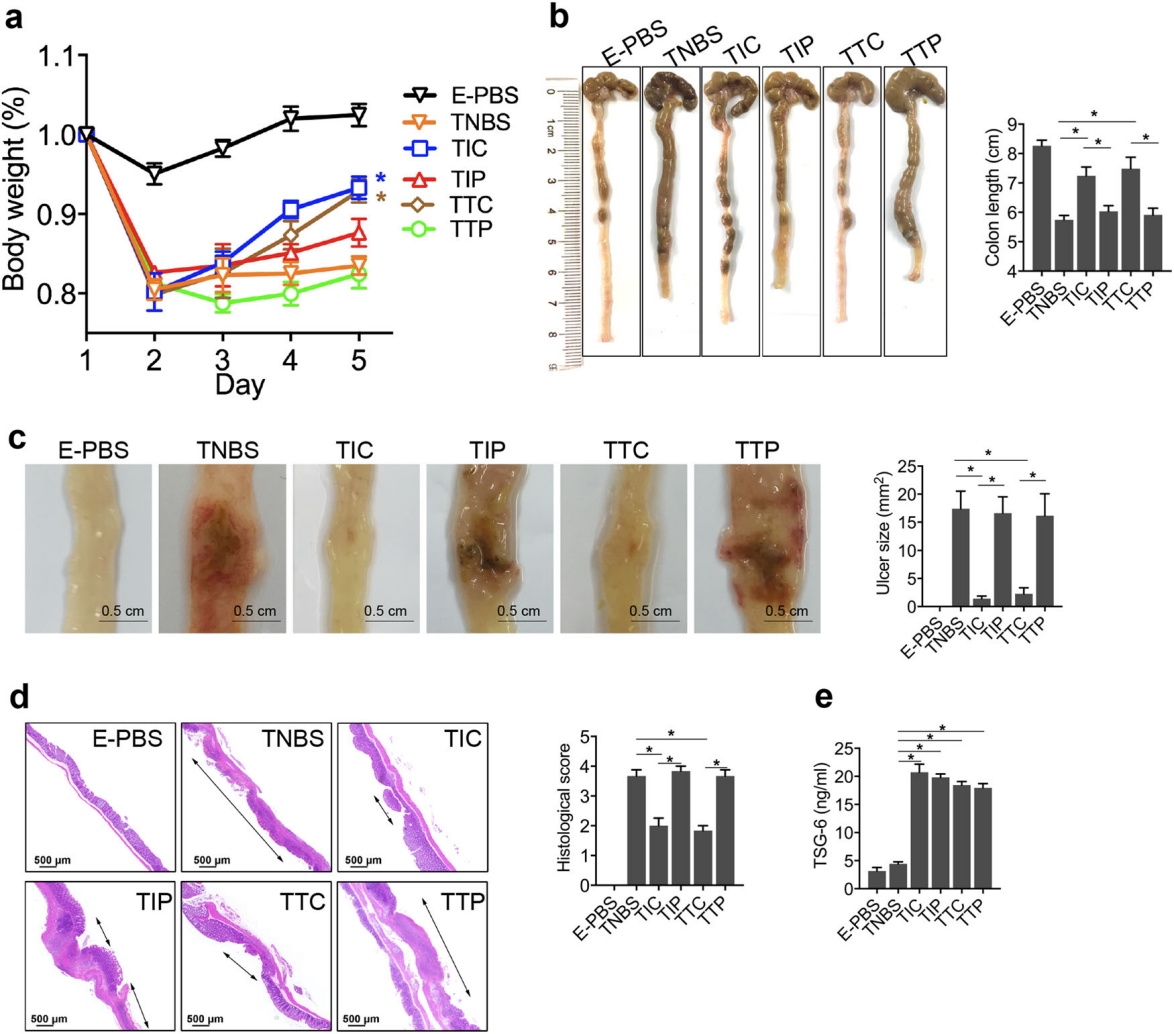
TSG-6 and iPSC-MSCs promote mucosal healing in murine colitis through interactions between CD44 and hyaluronic acid. On the next day after intrarectal administration of TNBS, mice received different treatment: colitic mice given an intraperitoneal injection of 2 × 10^6^ iPSC-MSCs/mouse after the pretreatment of intraperitoneal injections of CD44 inhibitory peptide (PEP-1) (TIP group) or control peptide (C-PEP) (TIC group) at the dose of 10 mg/kg daily; colitic mice treated with daily intraperitoneal injections of recombinant human TSG-6 (rhTSG6) at the dose of 5 μg/mouse after the pretreatment of intraperitoneal injections of PEP-1 (TTP group) or C-PEP (TTC group) at the dose of 10 mg/kg daily; healthy mice receiving daily intraperitoneal injections of PBS as a negative control (E-PBS group). **a** Changes of body weight were monitored daily during the whole observation. *P < 0.05 versus TNBS group. **b** Gross morphology and length of colons in individual groups. **c** Representative photographs of gross colonic ulcers are shown. Colonic ulcer area was measured by the ImageJ software. **d** Representative histology images and histological scores in individual groups. Double arrowhead lines indicated colonic ulcers. **e** Serum TSG-6 protein levels in individual groups were determined by ELISA. *n* = 6 mice per group. *P < 0.05

**Fig. 5:**
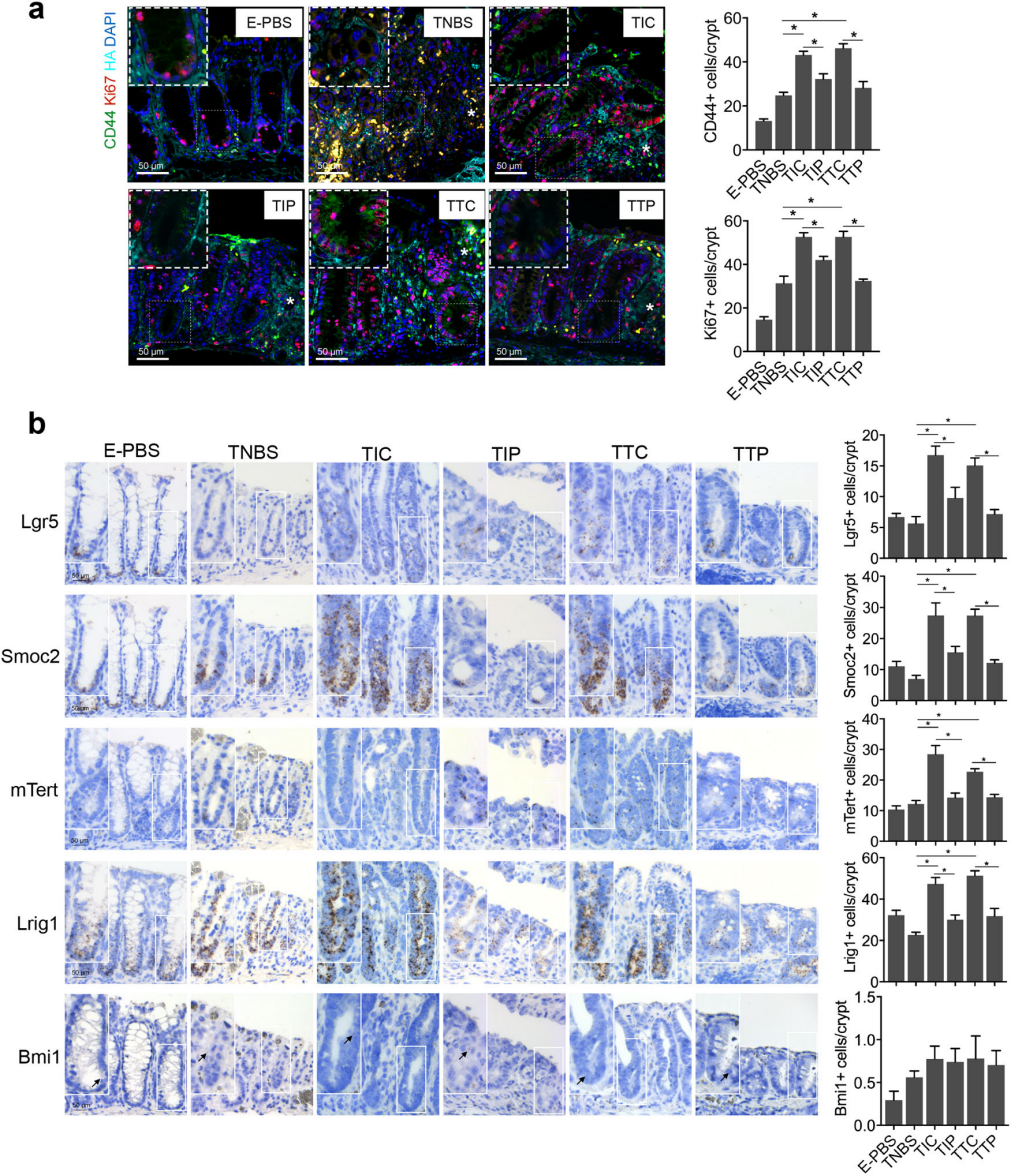
TSG-6 and iPSC-MSCs promote epithelial cell proliferation through interactions between CD44 and hyaluronic acid (HA). **a** Immunofluorescence staining for HA, Ki67, and CD44. Asterisks indicate ulcer margins. The numbers of Ki67+ cells or CD44+ cells were counted from five glands at ulcer margins. **b** Serial sections in situ hybridized for cycling crypt base columnar cell markers (Lgr5, Smoc2) and quiescent +4 stem cell markers (mTert, Lrig1, and Bmi1). Bar graph shows the numbers of positively stained cells of the indicated markers per crypt in each group. Arrows indicate Bmi1-positive cells in the crypt. *n* = 6 mice per group. *P < 0.05

To validate the mechanism by which iPSC-MSCs promote mucosal healing in murine colitis, recombinant human TSG-6 (rhTSG-6) was used to treat the experimental murine colitis model. The effects of rhTSG-6 treatment largely reproduced the therapeutic effects of iPSC-MSCs (Fig. 4a–d). Of note, rhTSG-6 treatment increased the numbers of CD44+ epithelial cells, Ki67+ epithelial cells, CBC stem cells (Lgr5+ and Smoc2+), and +4 stem cells (mTert+ and Lrig1+) (Fig. 5a, b).

To test the hypothesis that iPSC-MSCs and TSG-6 regulate epithelial cell proliferation through interactions between CD44 and HA during mucosal repair, colitic mice were injected with either PEP-1^33^, a peptide that specifically blocks interactions between CD44 and HA, or control peptide (C-PEP) after treatment with iPSC-MSCs or rhTSG-6. PEP-1 blocking of CD44 from interactions with HA abrogated the therapeutic effects of iPSC-MSCs or rhTSG-6, as shown by the relatively lower weights, shorter colons, and larger colonic ulcers in mice (Fig. 4a–d). Furthermore, PEP-1 treatment also decreased the number of Ki67+ epithelial cells, CD44+ epithelial cells, CBC stem cells (Lgr5+ and Smoc2+), and +4 stem cells (mTert+ and Lrig1+) (Fig. 5a, b). No significant differences in clinical parameters or histological manifestation were noted in colitic mice treated with either PEP-1 or C-PEP alone (data not shown). These data implied that iPSC-MSCs and TSG-6 promoted the proliferation of epithelial cells in colonic crypts through interactions between CD44 and HA.

### TSG-6-induced proliferation of colonoids ex vivo depends on interactions between CD44 and HA

**Fig. 6:**
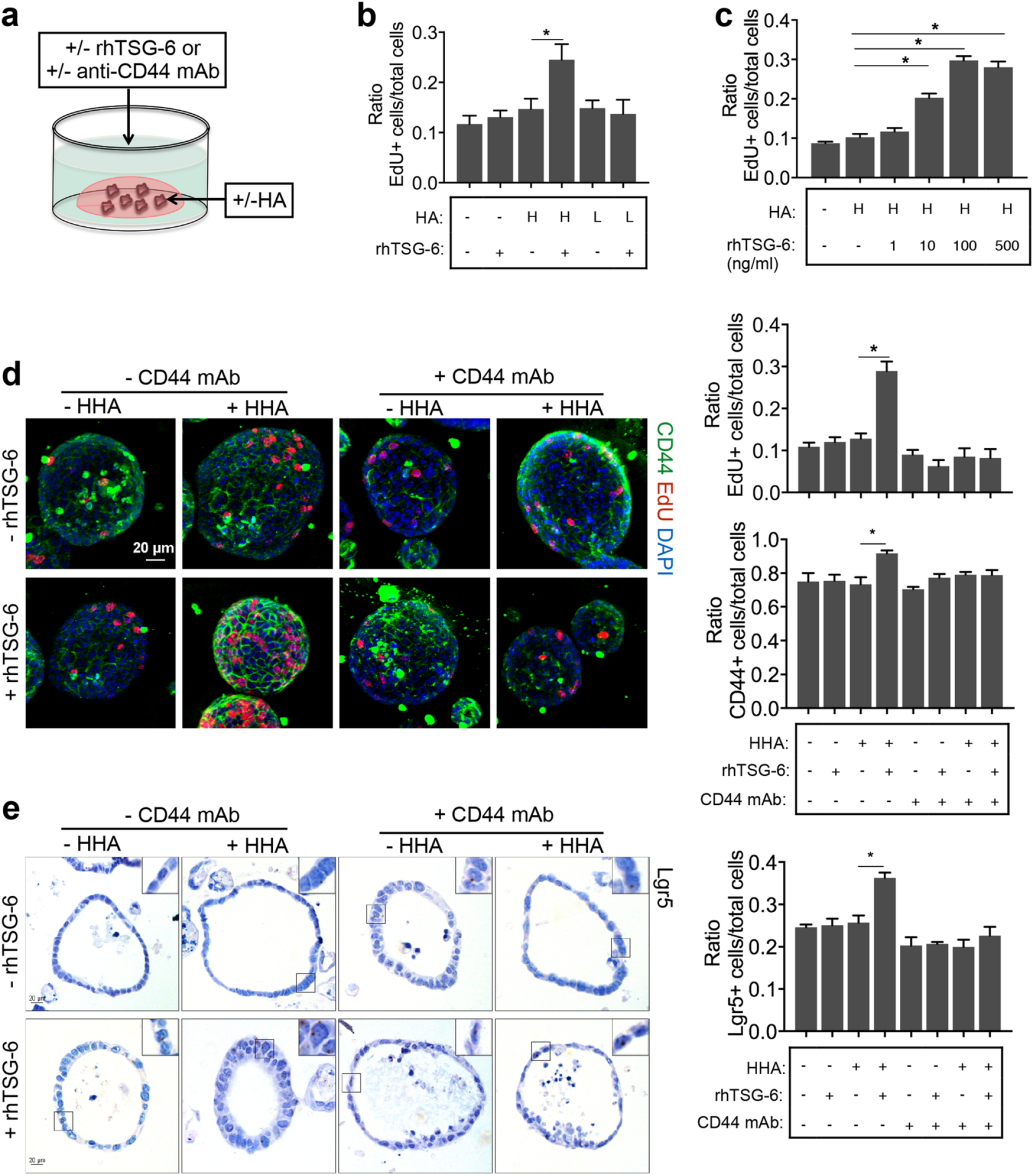
TSG-6-induced proliferation of colonoids ex vivo depends on interactions between CD44 and hyaluronic acid (HA). **a** Schematic diagram of organoid culture. **b** Murine colonoids were treated with 100 ng/mL recombinant human TSG-6 (rhTSG6) or PBS in the presence of or absence of 500 μg/mL recombinant human TSG-6 (rhTSG6) or PBS in the presence or absence of 500 μg/mL high molecular weight HA (HHA) or low molecular weight HA (LHA) for 16 h. Murine colonoids were immunostained for 5-ethynyl-20-deoxyuridine (EdU) and nucleus. **c** Murine colonoids were treated with different concentrations of rhTSG-6 in the presence of 500 μg/mL HHA, and colonoids were treated without rhTSG-6 or HHA were used as a control. **d** Murine colonoids were preincubated with 5 μg/mL anti-CD44 monoclonal antibody (anti-CD44 mAb, clone KM201) or rat isotype control antibody for 6 h following treatment with 100 ng/mL rhTSG-6 or PBS in the presence or absence of 500 μg/mL HHA for 16 h. Murine colonoids were immunostained for EdU (red), nucleus (blue), and CD44 (green). Total cell number, EdU-positive cells, and CD44-positive cells were counted and results were expressed as EdU-positive cells/total cells ratio or CD44-positive cells/total cells ratio. **e** RNA in situ hybridization for the intestinal epithelial stem cell marker (Lgr5) in murine colonoids. Total cell number and Lgr5-positive cells were counted and results were expressed as Lgr5-positive cells/total cells ratio. *n* = 4 independent experiments. *P < 0.05

To further validate the roles of TSG-6 in iPSC-MSCs in regulating epithelial cell proliferation, three-dimensional mouse or human organoids of colonic crypts were cultured (Fig. 6a). Murine colonoids were embedded in 10% HA/Matrigel or 10% PBS/Matrigel and then left untreated or incubated with rhTSG-6 for 16 h. Interestingly, incubation with rhTSG-6 led to a significant dose-dependent increase in the cell proliferation rate, as quantified by the EdU-labeled cell/total cell ratio, in murine colonoids grown in the presence of high molecular weight HA (HHA) rather than low molecular weight HA (LHA) (Fig. 6b–d). Furthermore, rhTSG-6 also increased the frequency of CD44+ cells and Lgr5+ cells in colonoids (Fig. 6d, e). The increased proliferation rate induced by rhTSG-6 in HHA-embedded murine colonoids decreased in response to anti-CD44 monoclonal antibody (anti-CD44 mAb, clone KM201), which was shown to specifically block interactions between CD44 and HA^34^ (Fig. 6d). The promotion effect of rhTSG-6 was similar to that observed in human colonic mucosa-derived organoids (Supplementary Fig. 7).

To further verify the role of CD44 in TSG-6-induced epithelial cell proliferation, murine colonoids were transfected with CD44 shRNA-expressing lentiviral vectors (Supplementary Fig. 8a). Knockdown of CD44 significantly suppressed rhTSG-6-induced epithelial cell proliferation and the increase in CD44+ cells and Lgr5+ cells in colonoids (Supplementary Fig. 8b, c).

Collectively, these data demonstrated that TSG-6-induced crypt epithelial cell proliferation was dependent on interactions between CD44 and HA.

### TSG-6 and iPSC-MSCs accelerate intestinal epithelial cell proliferation in an Akt-dependent manner

**Fig. 7:**
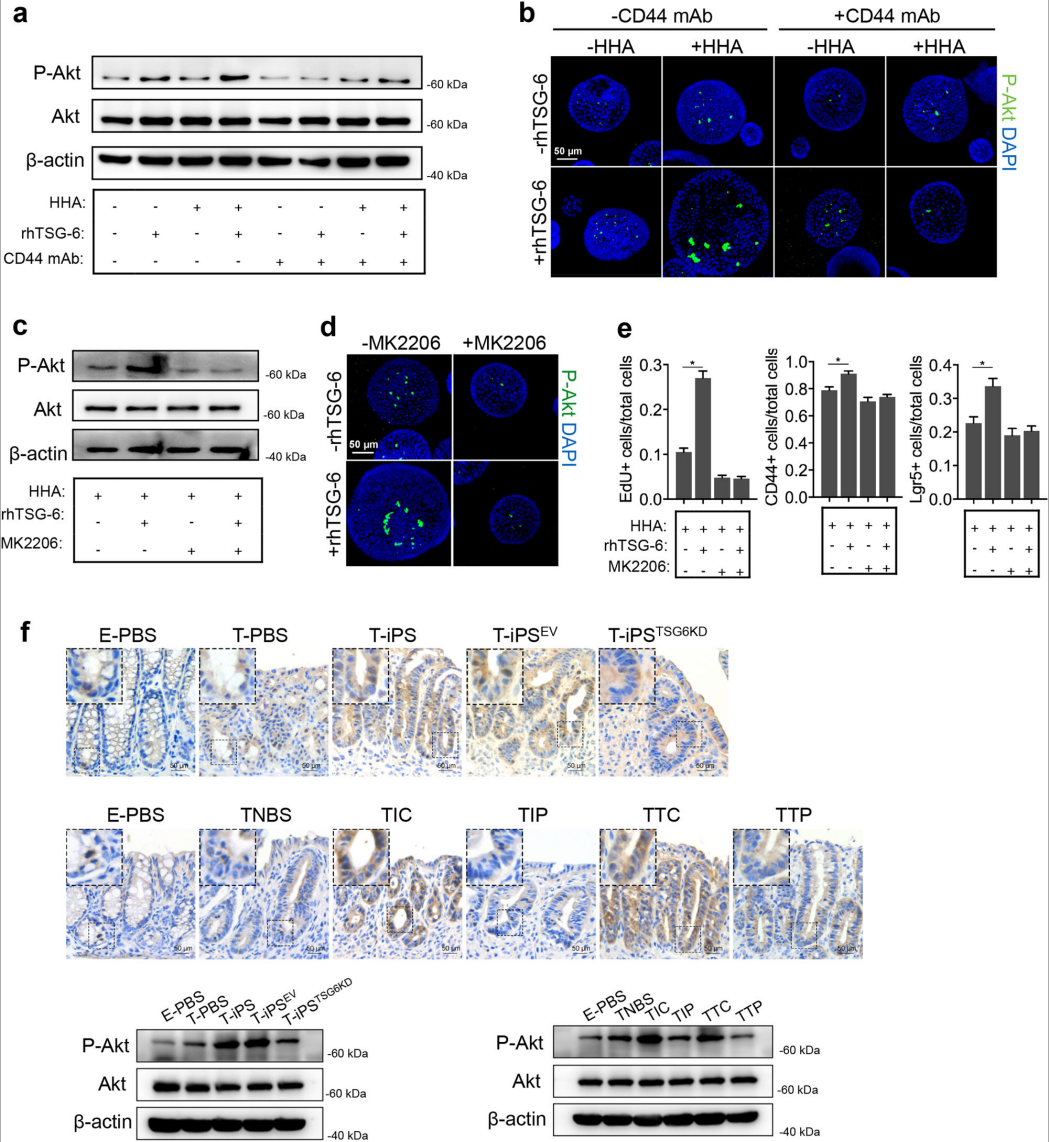
TSG-6 and iPSC-MSCs accelerate intestinal epithelial cell proliferation in an Akt-dependent manner. Murine colonoids were preincubated with 5 μg/mL anti-CD44 monoclonal antibody (anti-CD44 mAb, clone KM201) or rat isotype control antibody for 6 h following treatment with 100 ng/mL recombinant human TSG-6 (rhTSG6) or PBS in the presence or absence of 500 μg/mL high molecular weight HA (HHA) for 16 h. **a** Murine colonoids were collected for protein extraction and then immunoblotted with the indicated antibodies. **b** Murine colonoids in individual groups were subjected to anti-phospho-Akt immunofluorescence (anti-P-Akt) (green). **c**, **d** Murine colonoids were incubated with 100 ng/mL recombinant human TSG-6 (rhTSG-6) or PBS plus MK2206 (P-Akt specific inhibitor, 5 μM) or DMSO in the presence of 500 μg/mL HHA for 16 h. Murine colonoids in individual groups were harvested for western blot analyses or immunostained for P-Akt (green) and nucleus (blue). **e** Murine colonoids were immunostained with 5-ethynyl-20-deoxyuridine (EdU) and CD44 or in situ hybridized for intestinal epithelial stem cell marker (Lgr5). Total cell number, EdU-positive cells, CD44-positive cells, and Lgr5-positive cells were counted and results were expressed as EdU-positive cells/total cells ratio, CD44-positive cells/total cells ratio, and Lgr5-positive cells/total cells ratio. *n* = 4 independent experiments. *P < 0.05. **f** The protein expression of P-Akt in murine colon tissues from each group described in Figs. 1 and 4 was detected by immunohistochemical staining and western blot analyses

Previous studies showed that the phosphoinositide-3 kinase/Akt and mitogen-activated protein kinase (MAPK) pathways regulate HA-CD44-mediated cancer cell proliferation^35,36^. Western blot analyses revealed that rhTSG-6 treatment in the presence of HHA induced Akt activation in murine colonoids (Fig. 7a). However, rhTSG-6 treatment did not affect p44/p42 MAPK (extracellular signal–regulated kinase 1/2) activation (Supplementary Fig. 9). Immunofluorescence staining for Phospho-Akt (P-Akt) showed that, in the presence of HHA, rhTSG-6 enhanced Akt phosphorylation in murine colonoids (Fig. 7b). Not surprisingly, rhTSG-6-induced Akt phosphorylation was reduced by anti-CD44 mAb (Fig. 7a, b).

To verify that TSG-6 promoted epithelial cell proliferation via Akt activation, we tested whether MK2206, an Akt-specific inhibitor, could attenuate the effects of rhTSG-6. MK2206 decreased Akt phosphorylation and abrogated the promoting effect of rhTSG-6 on murine colonoid proliferation (Fig. 7c–e).

Immunohistochemistry and western blot analyses of P-Akt in murine colon tissues showed that iPSC-MSCs and rhTSG-6 increased Akt phosphorylation in colitic mice and that knockdown of TSG-6 in iPSC-MSCs or PEP-1 treatment suppressed this effect (Fig. 7f).

In summary, these in vivo and ex vivo experiments demonstrated that TSG-6 and iPSC-MSCs promoted crypt epithelial cell proliferation via Akt activation.

## Discussion

**Fig. 8:**
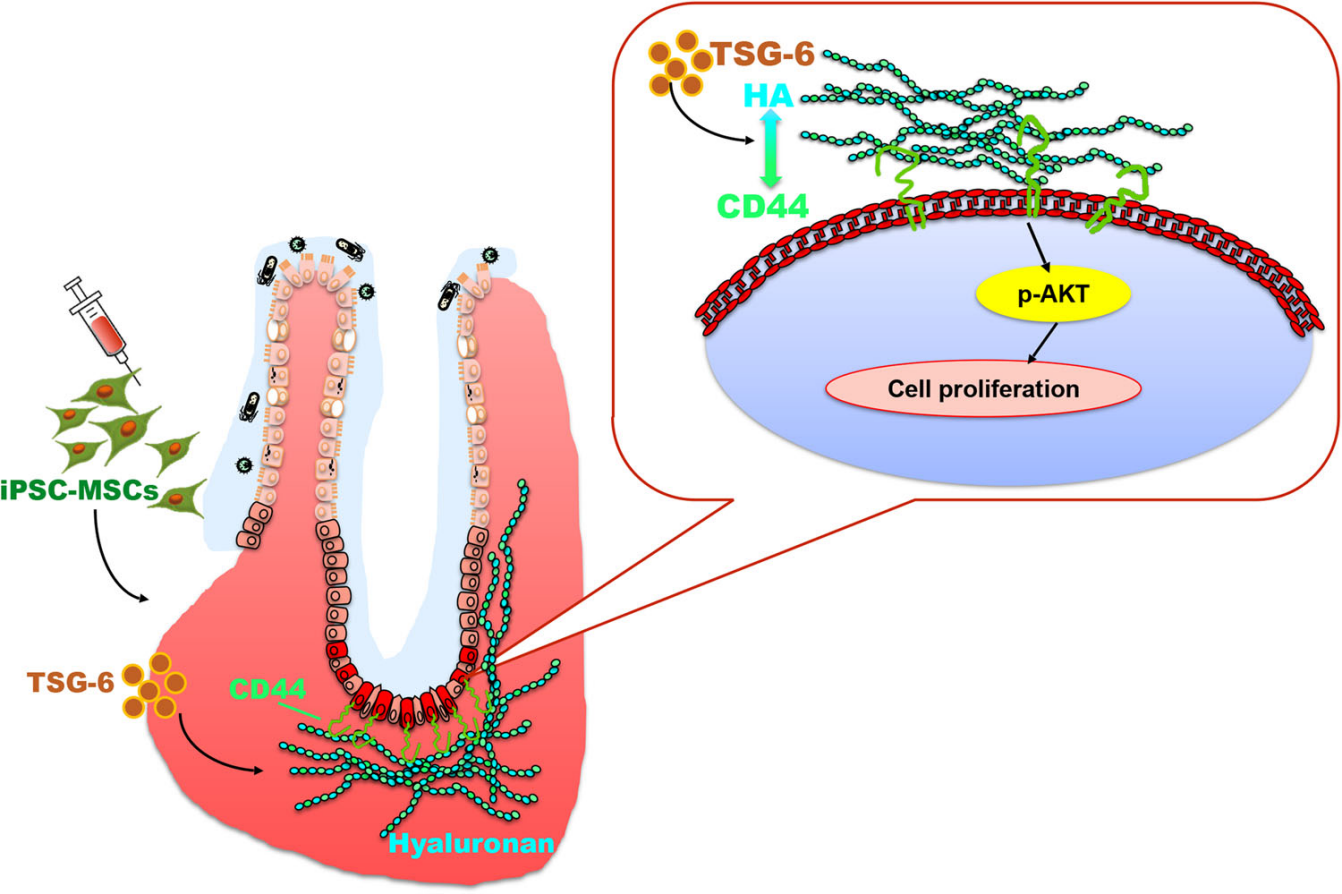
Model of the mechanisms by which iPSC-MSCs promote colonic mucosal regeneration via TSG-6. iPSC-MSC treatment promotes colonic epithelial proliferation to accelerate mucosal healing in mouse models of inflammatory bowel diseases via TSG-6. The effects of iPSC-MSCs is dependent on interactions between hyaluronic acid (HA) and CD44 in an Akt-dependent manner

Based on the unique capability of MSCs to promote tissue repair, MSC treatment is a promising strategy to improve mucosal healing in IBD patients. However, the limitations of expandability and difficulty in standardization hinder their application potential. In previous studies, we used human iPSCs to generate functional MSCs with the capacity to self-renew in culture for >120 population doublings^18–20^. Other studies, including ours, indicated that iPSC-MSCs offer a promising alternative to tissue-derived MSCs for therapeutic use, including treatment of limb ischemia and colitis^18,37^. In the present study, we showed that iPSC-MSCs promote intestinal repair in murine colitis, and we identified TSG-6, released by iPSC-MSCs, as a key factor necessary for iPSC-MSC promotion of colonic epithelial cell proliferation and wound repair after mucosal injury (Fig. 8).

In the current study, we first evaluated the therapeutic efficacy of iPSC-MSCs in both TNBS-induced and DSS-induced murine colitis models. Treatment with iPSC-MSCs improved murine colitis both clinically and histologically. Crypts of Lieberkuhn that harbor intestinal epithelial stem cells undergo proliferation and fission to heal mucosal injury in response to insults such as ischemia and inflammation^32^. Two stem cell populations have been implicated in epithelial regeneration: CBC stem cells, which cycle fast to maintain homeostasis, and +4 stem cells, which are quiescent in normal conditions but cycle fast to restore homeostasis after injury^32^. Our data indicated that iPSC-MSCs significantly promote the proliferation of colonic epithelial cells and expand the intestinal epithelial stem cell pool. Staining of serial sections showed that CBC stem cell markers (Lgr5 and Smoc2) share similar expression patterns, whereas +4 stem cell markers (mTert, Lrig1, and Bmi1) are expressed throughout crypts and, in some cases, overlap with that of Lgr5. Solid evidence has emerged that these three proposed +4 stem cell markers are robustly expressed in CBC cells, which casts doubts on the validity of these proteins as markers for +4 stem cells^31,38^. This difference in distribution may explain, in part, why not all the +4 stem cell markers (mTert, Lrig1, and Bmi1) showed significant differences after iPSC-MSC treatment. In contrast, Lgr5 and Smoc2 are shown to be exclusively expressed in CBC stem cells. Our data showed that iPSC-MSCs promote crypt epithelial cell proliferation and increase CBC stem cells in colitic mice.

The ex vivo murine colonoid and iPSC-MSC coculture system confirmed the notion that iPSC-MSCs promote the proliferation of colonic epithelial cells and increase CBC stem cells. Several studies have shown that MSCs stimulate intestinal stem cells to repair intestinal injuries either by induction of radiation or chemicals^39–41^. Most recently, Soontararak et al. reported that iPSC-MSCs stimulate intestinal epithelial cell proliferation and increase the numbers of Lgr5+ intestinal stem cells in a DSS-induced murine colitis model^37^, which is consistent with our data.

Many studies have demonstrated immunomodulatory effects of MSCs on immune cells^42^. Our previous study showed that iPSC-MSCs possess immunosuppressive properties and modulate allergic airway diseases and allergic rhinitis^19,20^. In the present study, the histological analysis indicated that iPSC-MSCs attenuate immune cell infiltration, and despite a severe inflammatory microenvironment, there were fewer Ki67+ epithelial cells in untreated colitic mice than in treated ones. Furthermore, this difference in Ki67+ epithelial cell numbers was apparent even when inflammation levels were comparable between the two groups. Collectively, these data expand the understanding that iPSC-MSCs stimulate intestinal epithelial proliferation to promote mucosal healing in colitic mice.

The mechanism underlying iPSC-MSCs-induced colonic epithelial cell proliferation remains unknown. Increasing evidence suggests that MSCs promote injured tissue recovery by secreting paracrine factors^43^. Our previous study also indicated that iPSC-MSCs promote vascular and muscle regeneration via paracrine mechanisms to attenuate limb ischemia in mice^18^. However, no specific factors have been identified as being responsible for the therapeutic effects of iPSC-MSCs in colitis. In the present study, the number of iPSC-MSCs detected was sustained in treated mice, especially in peritoneal cavities, throughout the observation period. In addition, we showed that TSG-6 released by iPSC-MSCs plays a key role in the tissue repair function of iPSC-MSCs. These data imply that iPSC-MSCs promote injured tissue recovery in a paracrine manner.

The synthesis and release of TSG-6 in iPSC-MSCs is conditioned by inflammatory mediators such as TNF-α, interleukin-1β (IL1β), and interferon (IFN)-γ. This indicates that iPSC-MSCs may sense and respond to an inflammatory environment whenever damage occurs. The in vivo and ex vivo experiments confirmed that TSG-6 plays an essential role in iPSC-MSC promotion of intestinal epithelial cell proliferation. This mechanism appears to be conserved in humans and mice. The therapeutic effects of daily intraperitoneal injections of rhTSG-6 for 4 days were similar to those of one single intraperitoneal injection of iPSC-MSCs. Further, the in vivo imaging analyses showed that the number of iPSC-MSCs in treated mice was almost sustained throughout the experiment. This suggests that iPSC-MSC function may be more long-lasting compared to the TSG-6 therapy alone. New therapeutic options with plasticity and sustained effects will be of great value, because they may allow long-term mucosal healing in IBD patients that prevent future disease complications.

Some studies have shown that TSG-6 activates limbal epithelial stem cells to promote diabetic corneal epithelial wound healing in stem cell niches^27,28^. However, the mechanism by which TSG-6 mediates intestinal epithelial cell proliferation has not been addressed until now. TSG-6 can directly modulate interactions between HA and cell surface receptor CD44^30^. Furthermore, HA expression increases in response to injury. CD44 is a marker of intestinal epithelial stem cells^32^. Here we showed in vivo and ex vivo that TSG-6 promotes colonic epithelial stem cell proliferation via CD44 and HA interactions. Furthermore, we demonstrated the Akt-dependent proliferative effects of TSG-6 by applying MK2206, an Akt-specific inhibitor, to colonoid cultures and confirmed this relationship by immunohistochemistry and western blot analyses of P-Akt in mouse colon tissues.

There were some limitations in this study. We observed that the effects of rhTSG-6 on colonic ulcer size tended to be slightly less than those of iPSC-MSCs. Furthermore, the number of proliferating epithelial cells in colonoids cocultured with iPSC-MSCs^TSG6KD^ was slightly higher than that in colonoids cultured without iPSC-MSCs but was lower than that with iPSC-MSCs expressing TSG-6. We also found that iPSC-MSCs^TSG6KD^ did not significantly increase the number of CD44+ and Lgr5+ cells in colonoids during our observation period. These results do not exclude the possibility that iPSC-MSCs secrete paracrine factors other than TSG-6. However, our present study showed that TSG-6 plays a key role in mediating the therapeutic effects of iPSC-MSCs. Although teratogenic effects of iPSC-MSCs were not observed in our laboratory and others^18,44^, safety concerns such as this should be addressed before clinical application. Recently, iPSC-based transplantation to treat age-related macular degeneration and Parkinson’s disease has been performed in patients, and no serious adverse events were noted^45,46^. With the advance of methodologies to induce iPSCs, additional clinical trials of iPSC-based transplantation should be conducted.

In conclusion, we showed that iPSC-MSCs stimulate colonic epithelial cell proliferation via TSG-6 to promote mucosal healing in murine colitis. We also revealed Akt-dependent interactions between the extracellular matrix (HA) and stem cell compartment (CD44+ cells) as a novel mechanism by which TSG-6 promotes crypt epithelial cell proliferation. Results of this study illuminate the potential for therapeutic use of iPSC-MSCs to treat IBD.

## Methods and materials

### Ethics statements

In this study, intestinal samples were obtained from IBD patients undergoing surgery at the First Affiliated Hospital of Sun Yat-sen University. All participants provided written informed consent. The study protocol was approved by the Human Ethics Committee of the First Affiliated Hospital of Sun Yat-sen University ([2018]103). The veterinary care of mice and the animal experiments were approved by the Animal Care and Medical Ethics Committee of the First Affiliated Hospital of Sun Yat-sen University ([2018]078).

### Animal experiments

BALB/c or C57BL/6J mice (6–8 weeks old) were purchased from Nanjing Biomedical Research Institute of Nanjing University (Nanjing, China) and were allowed to acclimatize for 1 week before the experiments began. The TNBS (Sigma-Aldrich, St. Louis, MO, USA) or DSS (MP Biomedicals, Solon, OH, USA) induced mouse models were prepared as previously described^47^. For the TNBS model, male mice received TNBS (2–3 mg/mouse) in 50% ethanol intrarectally, whereas control mice received 50% ethanol alone on day 1. On the next day, mice were injected intraperitoneally with iPSC-MSCs with or without TSG-6 knockdown (2 × 10^6^ cells/mouse) or PBS as a control. In other experiments, mice were injected intraperitoneally with one dose of iPSC-MSCs (2 × 10^6^ cells/mouse), rhTSG-6 (5 μg/mouse/day × 4 days), or PBS starting on day 2 following daily intraperitoneal injection of PEP-1 or a control peptide (10 mg/kg/day × 4 days). For the DSS model, female C57BL/6J mice were treated with water containing 3% w/v DSS ad libitum for 1 week or water as control. On day 6, one dose of iPSC-MSCs (2 × 10^6^ cells/mouse) with or without TSG-6 knockdown was injected intraperitoneally into mice. PBS was used as a control. Body weight was monitored daily. At the end of the experiment, mice were sacrificed for sample collection. Blood was collected by cardiac puncture and centrifuged at 3000 rpm for 15 min at 4 °C. Plasma samples were assessed for TSG-6 levels by enzyme-linked immunosorbent assay (ELISA). Colons were removed and then opened longitudinally, and colonic ulcer severity was evaluated under a stereomicroscope after colon lengths were measured. Colon lengths and ulcer sizes were analyzed using the ImageJ software (National Institutions of Health, USA). Colon samples were snap-frozen in liquid nitrogen and stored at −80 °C. Some of these samples were fixed in neutral buffered 10% formalin overnight before they were embedded in paraffin. For the histological evaluation, tissue sections were deparaffinized in xylene and hydrated through a graded series of alcohol to water. Sections were stained with hematoxylin and eosin (H&E), and H&E images were obtained with a Zeiss AxioScan Z1 (Carl Zeiss, Jena, Germany).

### Culture of human iPSC-MSCs and TSG-6 knockdown in iPSC-MSCs

Human iPSC-MSCs were prepared as we previously reported^18–20^. Briefly, the iPSC-MSCs were generated from iPSC-iMR90-5 (WiCell Research Institute, Madison, WI, USA). MSCs were induced from iPSCs as previously described^48^. MSCs were collected by sorting for CD105+/CD24− cells that were then cultured in 90% KnockOut Dulbecco’s modified Eagle’s medium (DMEM; Gibco, Grand Island, NY, USA) supplemented with 10% serum replacement medium (Gibco) and basic fibroblast growth factor (10 ng/mL, Gibco). MSCs were identified by examining their expression of CD34, CD24, CD44, CD31, CD73, CD29, CD166, and CD105 cell surface markers using fluorescein isothiocyanate- or phycoerythrin-labeled antibodies (BD Biosciences, San Jose, CA, USA), because MSCs express CD44, CD73, CD105, and CD166, and lack expression of CD45, CD34, and CD133. MSCs at passages 4–9 were used in the experiments.

iPSC-MSCs were plated in six-well plates. At 80% confluence, iPSC-MSCs were treated with different concentrations of inflammatory cytokines, including recombinant human TNF-α (Peprotech, Rocky Hill, NJ, USA), recombinant human IFN-γ (Peprotech), and recombinant human IL1β (Peprotech), for 16 h. Cell supernatants were collected to measure TSG-6 levels by ELISA, and then cells were harvested in RIPA medium for protein extraction.

GV248-GFP-shRNA-TSG-6 lentivirus vectors, purchased from Shanghai GeneChem Co. (Shanghai, China), had shRNA sequences of CTAAGGGCAGAGTTGGATA. Lentiviruses containing shRNA targeting TSG-6 was transfected into iPSC-MSCs according to the manufacturer’s instructions. iPSC-MSCs transfected with GV248-GFP empty lentivirus vectors were used as a control. Puromycin (2 μg/mL) was used to select stable clones. Transfection efficiency was assessed under a fluorescence microscope, and TSG-6 expression levels were determined by quantitative real-time reverse transcriptase polymerase chain reaction (qRT-PCR) and western blot analyses.

### Enzyme-linked immunosorbent assay

TSG-6 levels in serum or cell culture supernatants were determined by ELISA (Raybiotech, Norcross, GA, USA) according to the manufacturer’s instructions. Each sample and a standard protein was added to a single well of an ELISA plate and incubated at room temperature (RT) for 2.5 h. After washing, 100 μL of biotin antibody was added to each well, and plates were incubated at RT for 1 h. Then streptavidin solution was introduced for incubation at RT for an additional 45 min, followed by 100 μL of 3,3′,5,5′-tetramethylbenzidine substrate and incubation at RT for 30 min in the dark until stop solution was added. The optical density of each well was immediately determined using a microplate reader set to 450 nm. The concentration of serum TSG-6 was calculated based on a standard curve.

### RNA extraction and qRT-PCR

Total RNA was extracted from colon samples or cells using TRIzol Reagent (Invitrogen, Carlsbad, CA, USA) according to the manufacturer’s instructions. A Transcriptor First Strand cDNA Synthesis Kit (Roche, Basel, Switzerland) was used to synthesize cDNA, and qRT-PCR was performed using Fast Start Universal SYBR Green Master (Roche). Primer sequences are as following: human Cd44 (forward primer, CTGCCGCTTTGCAGGTGTA; reverse primer, CATTGTGGGCAAGGTGCTATT), human Tsg6 (forward primer, TCATGTCTGTGCTGCTGGATG; reverse primer, GGGCCCTGGCTTCACAA), and human glyceraldehyde 3-phosphate dehydrogenase (Gapdh) (forward primer, GACCTGCCGTCTAGAAAAACC; reverse primer, GCTGTAGCCAAATTCGTTGTC). The expression of each target mRNA normalized to Gapdh was determined. A comparative threshold cycle method was used to compare the expression in the treatment and control groups.

### Western blot analysis

Total protein was extracted for western blot analysis using RIPA lysis and extraction buffer (Thermo Fisher Scientific, Rockford, IL, USA) supplemented with 1× protease and phosphatase inhibitor cocktail (Cell Signaling Technology, Beverly, MA, USA). The proteins were boiled at 95 °C for 5 min and then processed for western blotting as previously described^49^. Briefly, proteins were separated on sodium dodecyl sulfate-polyacrylamide gels and blotted on nitrocellulose membranes that were incubated in 5% bovine serum albumin (BSA)/Tris-buffered saline and Tween 20 after incubation overnight at 4 °C with primary antibodies against TSG-6 (R&D, Minneapolis, MN, USA, 0.2 μg/mL), Akt (1:1000), P-Akt (Ser473) (1:1000), p44/42 MAPK (1:1000), phosphate p44/42 MAPK (1:1000), GAPDH (1:1000), or β-actin (1:1000). The antibodies were purchased from Cell Signaling Technology unless otherwise noted. After washing, the blots were incubated at RT for 1 h with anti-rabbit or anti-goat IgG horse radish peroxidase (HRP)-linked antibody (Abcam, Cambridge, UK, 1:2000).

### Immunocytochemistry

Cells were fixed with 4% paraformaldehyde at RT for 20 min and then incubated first with blocking buffer (3% BSA in 0.5% Triton X-100 in PBS) at RT for 1 h and second with primary antibody against TSG-6 (R&D, 10 μg/mL) at 4 °C overnight. After washing, cells were incubated with Alexa Fluor 594 conjugated anti-goat IgG antibody (Invitrogen) at RT for 1 h, followed by counterstaining with bis-benzamide (Hoechst 33258, Invitrogen). Images were obtained using an LSM 780 confocal microscope (Carl Zeiss).

### Immunofluorescence and immunohistochemistry

Tissue sections were deparaffinized and then hydrated through a graded series of alcohol to water. Antigen was retrieved with citrate buffer (pH 6.0). For the immunohistochemical analysis, sections were incubated with 3% H_2_O_2_ in PBS for 10 min. Sections were then incubated with blocking buffer (3% BSA in 0.5% Tween-20 in PBS) at RT for 1 h, followed by incubation at 4 °C overnight with primary antibodies against Ki67 (Abcam, 1:200), CD44 (Santa Cruz Biotechnology, Santa Cruz, CA, USA, 1:50), HRP-conjugated HA-binding protein (Millipore, Billerica, MA, USA, 200 μg/mL), TSG-6 (R&D, 10 μg/mL), GFP (Cell Signaling Technology, 1:100), or P-Akt (Ser473) (Cell Signaling Technology, 1:100). After washing, sections were incubated with Alexa Fluor 488 and 594-labeled secondary antibodies (Invitrogen), or Alexa Fluor 647 labeled streptavidin (Invitrogen) at RT for 1 h and then mounted using Fluoroshield (Sigma-Aldrich). Images were obtained with a Leica DM6B fluorescence microscope (Leica, Bensheim, Germany). For the immunohistochemistry analysis, sections were incubated with HRP-labeled streptavidin (Invitrogen) or HRP-labeled secondary antibody (Cell Signaling Technology) at RT for 30 min, and then color was developed using diaminobenzidine substrate. Finally, the sections were counterstained with hematoxylin and mounted using neutral balsam. Images were obtained under a Leica DMI1 microscope.

### RNA in situ hybridization

ISH was conducted using an RNAscope FFPE Assay Kit (Advanced Cell Diagnostics, Hayward, CA, USA) according to the manufacturer’s instructions. Briefly, tissue sections were boiled in target retrieval solution, pretreated with a protease, and then allowed to hybridize with probes (Lgr5, Smoc2, mTert, Bmi1, and Lrig1 for mouse samples, and Lgr5 for human samples). All probes were purchased from Advanced Cell Diagnostics. A signal amplification system (Amp1–6) was used before color development with diaminobenzidine substrate. Finally, the sections were counterstained with hematoxylin and mounted with neutral balsam.

### Generation and culture of mouse- or human mucosa-derived organoids

Organoids were prepared as we previously reported^49^. In brief, after several washes with Dulbecco’s PBS, samples were minced and incubated with 2 mM ethylenediaminetetraacetic acid buffer for 30–50 min at 4 °C under rotation. Then the samples were vigorously shaken or pipetted to release single crypts, followed by centrifugation 2–3 times with 150–200 × *g* for 5 min. Finally, the isolated crypts were embedded in Matrigel (Corning Lifescience, Acton, MA, USA) and overlaid with stem cell medium. For mouse colonic mucosa-derived organoids, a stem cell medium of Advanced DMEM/F12 (Invitrogen) supplemented with 2 mM GlutaMax (Invitrogen), 10 mM HEPES (Invitrogen), 1 × penicillin/streptomycin (Invitrogen), 1 × N2 (Invitrogen), 1 × B27 (Invitrogen), 1 mM *N*-acetyl-l-cysteine (Invitrogen), 50% Wnt3a-conditioned medium, 20% RSPO1-conditioned medium, 50 ng/mL mouse epidermal growth factor (EGF; Peprotech), and 100 ng/mL mouse Noggin (Peprotech) was used. Wnt3a- and RSPO1-conditioned media were collected as we previously described^32^. For human intestinal mucosa-derived organoids, a stem cell medium of Advanced DMEM/F12 supplemented with 2 mM GlutaMax, 10 mM HEPES, 1 × penicillin/streptomycin, 1 × N2, 1 × B27, 10 mM nicotinamide (Sigma-Aldrich), 1 mM *N*-acetyl-l-cysteine (Sigma-Aldrich), 50% Wnt3a-conditioned medium, 20% RSPO1-conditioned medium, 50 ng/mL human EGF (Peprotech), 100 ng/mL human Noggin (Peprotech), 10 μM SB202190 (Selleck, Houston, TX, USA), 0.5 μM A8301 (Sigma-Aldrich), and 10 nM [Leu15]-gastrin I (Sigma-Aldrich). The medium was replaced every 2 days. Growth of organoids was observed under an inverted phase-contrast microscope. For all the experiments, organoids at passages 2–5 were used. Human- or mouse-derived intestinal organoids embedded in Matrigel with or without 500 μg/mL HA were plated on microplates. CD44 is a Wnt target gene; therefore, to avoid Wnt effects on CD44, on days 4–6 after plating, organoids were incubated with Wnt-free medium for 24 h followed by pretreatment with anti-CD44 monoclonal antibody (KM201, 5 μg/mL; Abcam or Hermes-1, 5 μg/mL, Invitrogen) or rat isotype control antibody (5 μg/mL, Invitrogen) for 6 h, followed by treatment with rhTSG-6 for 16 h.

### Coculture of colonoids and iPSC-MSCs

At 80% confluence, iPSC-MSCs with or without TSG-6 knockdown in the bottom chambers of a Transwell plate were activated with 100 ng/mL TNF-α for 16 h before use. Mouse-derived colonoids embedded in Matrigel with or without 500 μg/mL HA were plated on the inner chamber of polyester Transwell inserts (0.4-μm pore size, Corning Lifescience). On days 4–6 after plating, murine colonoids were incubated with Wnt-free medium for 24 h, followed by co-culture with iPSC-MSCs in Wnt-free medium for 16 h.

### EdU staining

EdU staining was conducted as we previously described^49^. Organoids were incubated in 5 μm EdU in DMEM (Invitrogen) for 1 h at 37 °C and then fixed for 15 min with 3% formalin at RT, permeabilized with 0.5% Triton X-100 in PBS for 20 min, and incubated with a Click-iT reaction cocktail (Invitrogen) at RT for 30 min. After washing with PBS, organoids were incubated with Hoechst 33342 (Invitrogen, 1:1000). Immunofluorescence images were obtained using a Zeiss LSM780 confocal microscope, and a *Z*-stack series of confocal images was reconstituted using the Zeiss LSM780 software. Total cells and EdU-labeled nuclei were counted using the ImageJ software and expressed as the ratio of EdU-positive cells versus total cells.

### Whole-mount staining

Organoids were fixed with 10% formalin at RT for 15 min, permeabilized with 0.5% Triton-X in PBS for 20 min, blocked with 3% BSA in PBS for 1 h, and then incubated with antibodies against CD44 (Santa Cruz Biotechnology, 1:50) or P-Akt (Ser473) (Cell Signaling Technology, 1:100) overnight at 4 °C. After washing, organoids were incubated with secondary antibodies (Invitrogen, 1:400) overnight at 4 °C and then incubated with Hoechst 33342 (Invitrogen, 1:1000) for 30 min. Images were obtained via *Z*-reconstruction using a Zeiss LSM780 confocal microscope.

### Statistical analysis

Data were presented as means ± SEM. The significance of differences were tested by either a one-way analysis of variance test with Turkey’s post hoc test or Kruskal–Wallis test with Dunn’s post hoc test using the commercially available software (GraphPad, Prism, San Diego, CA, USA). *P* values of <0.05 were considered statistically significant.

## Supplementary information

Supplementary figure legends

Supplementary figure 1

Supplementary figure 2

Supplementary figure 3

Supplementary figure 4

Supplementary figure 5

Supplementary figure 6

Supplementary figure 7

Supplementary figure 8

Supplementary figure 9
