# Supplementary material for: Human induced pluripotent stem cell-derived mesenchymal stem cells promote healing via TNF-α-stimulated gene-6 in inflammatory bowel disease models
Source: Cell Death Dis. 2019 Sep 26;10(10):718. doi: 10.1038/s41419-019-1957-7 (PMC6763610; doi:10.1038/s41419-019-1957-7)
Supplement: Supplementary file 1 — Supplementary figure legends [file 41419_2019_1957_MOESM1_ESM.docx]

**Supplementary Data**.

**Supplementary figure 1. The *in vivo* tracing of iPSC-MSCs.** TNBS-induced colitic mice and EtOH-treated control mice received an intraperitoneal injection of 2 × 10^6^ luciferase-labeled or green fluorescent protein (GFP)-labeled iPSC-MSCs on day 2. (a) The *in vivo* tracing of luciferase-labeled iPSC-MSCs was monitored by the imaging system on day 2, day 3 and day 5. (b) The GFP-labeled iPSC-MSCs were detected by the flow cytometry. (c) Results of the flow cytometry analyses were expressed as GFP-positive cells/total cells ratio. n = 3 mice per group. **P* < 0.05. (d) The immunofluorescence stain for GFP and TSG-6 showed that GFP-positive cells engrafted into colonic mucosa, expressing TSG-6. PLF: peritoneal lavage fluid.

**Supplementary figure 2.** **The knockdown of TSG-6 in iPSC-MSCs.** The iPSC-MSCs were transfected with a lentivirus containing small hairpin (shRNA) targeting TSG-6 (iPSC-MSCs^TSG6KD^) or empty vector (iPSC-MSCs^EV^). (a) Transduction efficiency was tested and validated by immunofluorescent microscopy. iPSC-MSCs activated by 100 ng/ml TNFα for 16 hours were collected for qRT-PCR (b) and western blot analyses (c), and cell supernatant was collected for ELISA for TSG-6 (d). n = 3 independent experiments. **P* < 0.05.

**Supplementary figure 3. iPSC-MSCs promote mucosal healing via TSG-6 in dextran sulfate sodium (DSS)-induced colitis model.** DSS-treated colitic mice received intraperitoneal injections with 2 x 10^6^ iPSC-MSCs (D-iPS group), iPSC-MSCs^EV^ (D-iPS^EV^ group), iPSC-MSCs^TSG6KD^ (D-iPS^TSG6KD^ group), or PBS (D-PBS group), while water-induced control mice were given an injection of PBS as a negative control (W-PBS group). (a) Changes of body weight were monitored daily during the whole observation. (b) Gross morphology and length of colons in individual groups. **P* < 0.05. (c) Representative histology images in individual groups. Double arrowhead lines indicated colonic ulcers. (d) Colonic ulcer area was measured by ImageJ software. (e) Histological scores in individual groups. (f) Serum TSG-6 protein levels in individual groups were determined by ELISA. n = 6 mice for W-PBS group, n = 10 mice/group for the other groups. * *P* < 0.05.

**Supplementary figure 4. Hyaluronic acid (HA) expression in colitic mice.** On the next day after intrarectal administration of TNBS, mice received intraperitoneal injections with 2x 10^6^ iPSC-MSCs (T-iPS group), iPSC-MSCs^EV^ (T-iPS^EV^ group), iPSC-MSCs^TSG6KD^ (T-iPS^TSG6KD^ group), or PBS (T-PBS group), whereas healthy mice were given an injection of PBS as a negative control (E-PBS group). (a) Immunohistochemistry for HA-binding protein in individual groups showed increased distribution of HA in colitis. (b) Tissue sections were preincubated with PBS or HA (negative control) before incubation with HRP-labeled HA-binding protein.

**Supplementary figure 5.** On the next day after intrarectal administration of TNBS (2 mg/mouse), mice received intraperitoneal injections with 2 x 10^6^ iPSC-MSCs (T-iPS group), iPSC-MSCs^EV^ (T-iPS^EV^ group), iPSC-MSCs^TSG6KD^ (T-iPS^TSG6KD^ group), or PBS (T-PBS group). (a) At day 3 there was mild inflammation with comparable histological scores in individual groups. (b) Immunofluorescent staining for Ki67 and CD44. The numbers of Ki67-positive and CD44-positive cells were counted. n = 5 mice per group. **P* < 0.05.

**Supplementary figure 6. The increased expression and secretion of TSG-6 in iPSC-MSCs in respond to inflammatory cytokines.** (a) iPSC-MSCs were incubated with different concentrations of inflammatory cytokines (TNF-α, IFN-γ or IL1β) for 16 hours and harvested for western blot analyses with indicated antibodies. (b) iPSC-MSCs were treated with 100 ng/ml TNF-α, 100 ng/ml IFN-γ or 10 ng/ml IL1β for 16 hours. Cells were fixed, permeabilized and stained for the confocal microscopy. (c) After iPSC-MSCs were treated with 100 ng/ml TNF-α, 100 ng/ml IFN-γ or 10 ng/ml IL1β for 16 hours, the cell supernatant was collected for detection of TSG-6 protein levels by ELISA. n = 3 individual experiments. **P* < 0.05.

**Supplementary figure 7. TSG-6 accelerates the proliferation of human colonoids *ex vivo* dependent on interactions between CD44 and hyaluronic acid (HA).** (a) Human colonoids were preincubated with 5 μg/mL anti-CD44 monoclonal antibody (anti-CD44 mAb, clone Hermes-1) or rat isotype control antibody for 6 hours following treatment with 100 ng/ml recombinant human TSG-6 (rhTSG6) or PBS in the presence of or absence of 500 μg/ml high molecular weight HA (HHA) for 16 hours. Human colonoids were immunostained for 5-ethynyl-20-deoxyuridine (EdU) (red), CD44 (green), and nucleus (blue). Total cells number, EdU-positive cells and CD44-positive cells were counted and results were showed as EdU-positive cells/total cells ratio or CD44-positive cells/total cells ratio. (b) RNA in situ hybridization for the intestinal epithelial stem cell marker (Lgr5) in human colonoids. Total cell number and Lgr5-positive cells were counted and results were expressed as Lgr5-positive cells/total cells ratio. n = 4 independent experiments. **P* < 0.05.

**Supplementary figure 8.** **TSG-6 promotes the proliferation of colonoids *ex vivo* in a CD44-dependent manner.** (a) Murine colonoids were transfected with a lentivirus containing small hairpin RNA (shRNA) targeting CD44 (CD44KD) or empty vector (EV) as a control. Transduction efficiency was tested and confirmed by qRT-PCR. n = 3 independent experiments. **P* < 0.05. (b) Control and CD44 knockdown colonoids treated with or without recombinant human TSG-6 (rhTSG6) in the presence of or absence of high molecular weight hyaluronic acid (HHA) were immunostained for EdU (red), GFP (green) and nucleus (blue). Colonoids were immunostained for 5-ethynyl-20-deoxyuridine (EdU) (red) and nucleus (blue). Total cells and EdU-positive cells were counted and results were showed as EdU-positive cells/total cells ratio. (c) RNA in situ hybridization for the intestinal epithelial stem cell marker (Lgr5) in murine colonoids. Total cell number and Lgr5-positive cells were counted and results were expressed as Lgr5-positive cells/total cells ratio. n = 4 independent experiments. * *P* < 0.05.

**Supplementary figure 9.** **TSG-6 treatment did not affect the p44/p42 MAPK (ERK1/2) activation.** Murine colonoids were preincubated with 5 μg/ml anti-CD44 monoclonal antibody (anti-CD44 mAb, clone KM201) or rat isotype control antibody for 6 hours following treatment with 100 ng/ml recombinant human TSG-6 (rhTSG6) or PBS in the presence of or absence of 500 μg/ml high molecular weight hyaluronic acid (HHA) for 16 hours. Murine colonoids were collected for protein extraction and then immunobloted with indicated antibodies.
