# Supplementary figures and images for: Human induced pluripotent stem cell-derived mesenchymal stem cells promote healing via TNF-α-stimulated gene-6 in inflammatory bowel disease models
Source: Cell Death Dis. 2019 Sep 26;10(10):718. doi: 10.1038/s41419-019-1957-7 (PMC6763610; doi:10.1038/s41419-019-1957-7)

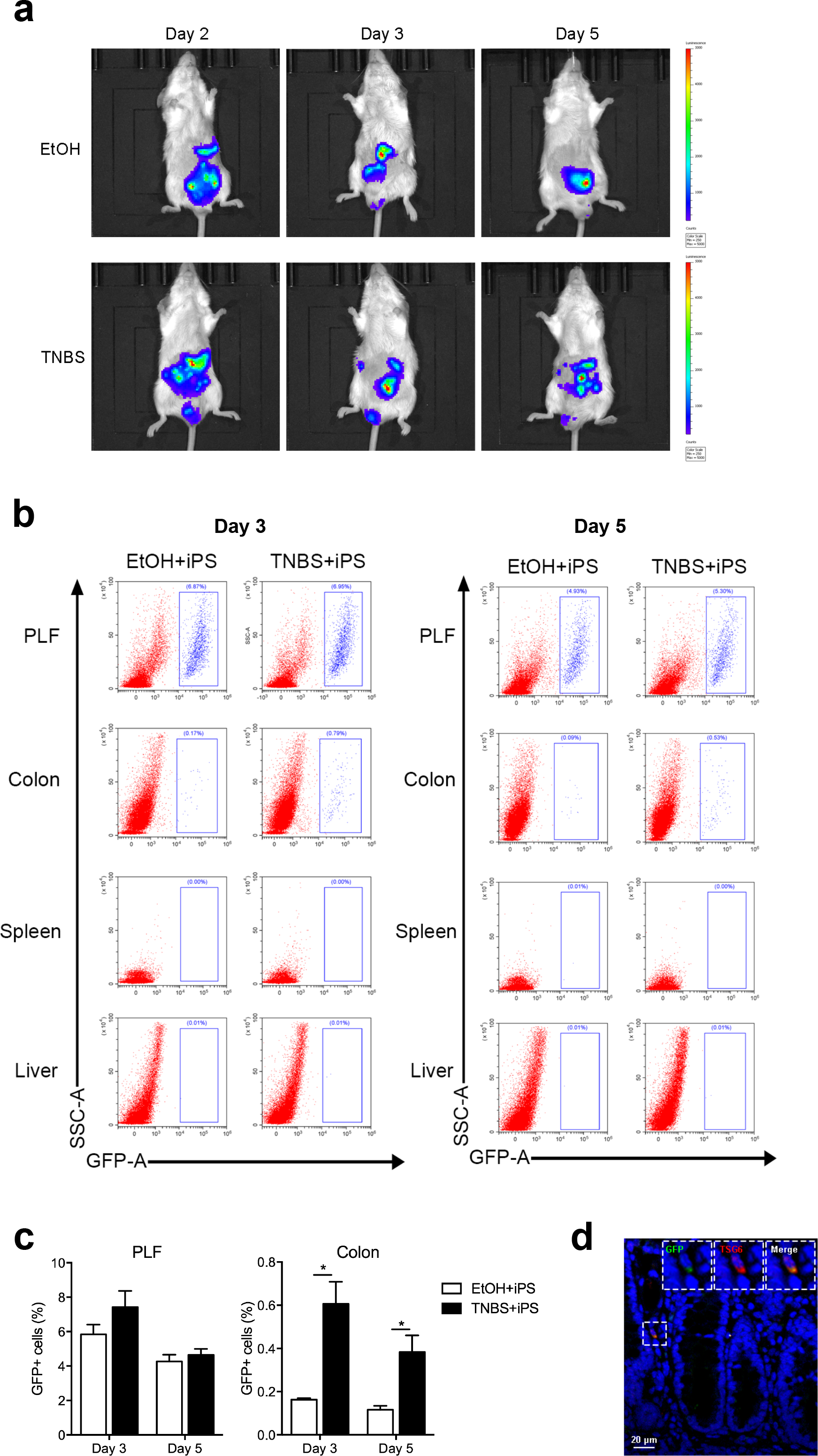

Supplement: Supplementary file 2 — Supplementary figure 1 [file 41419_2019_1957_MOESM2_ESM.tif]

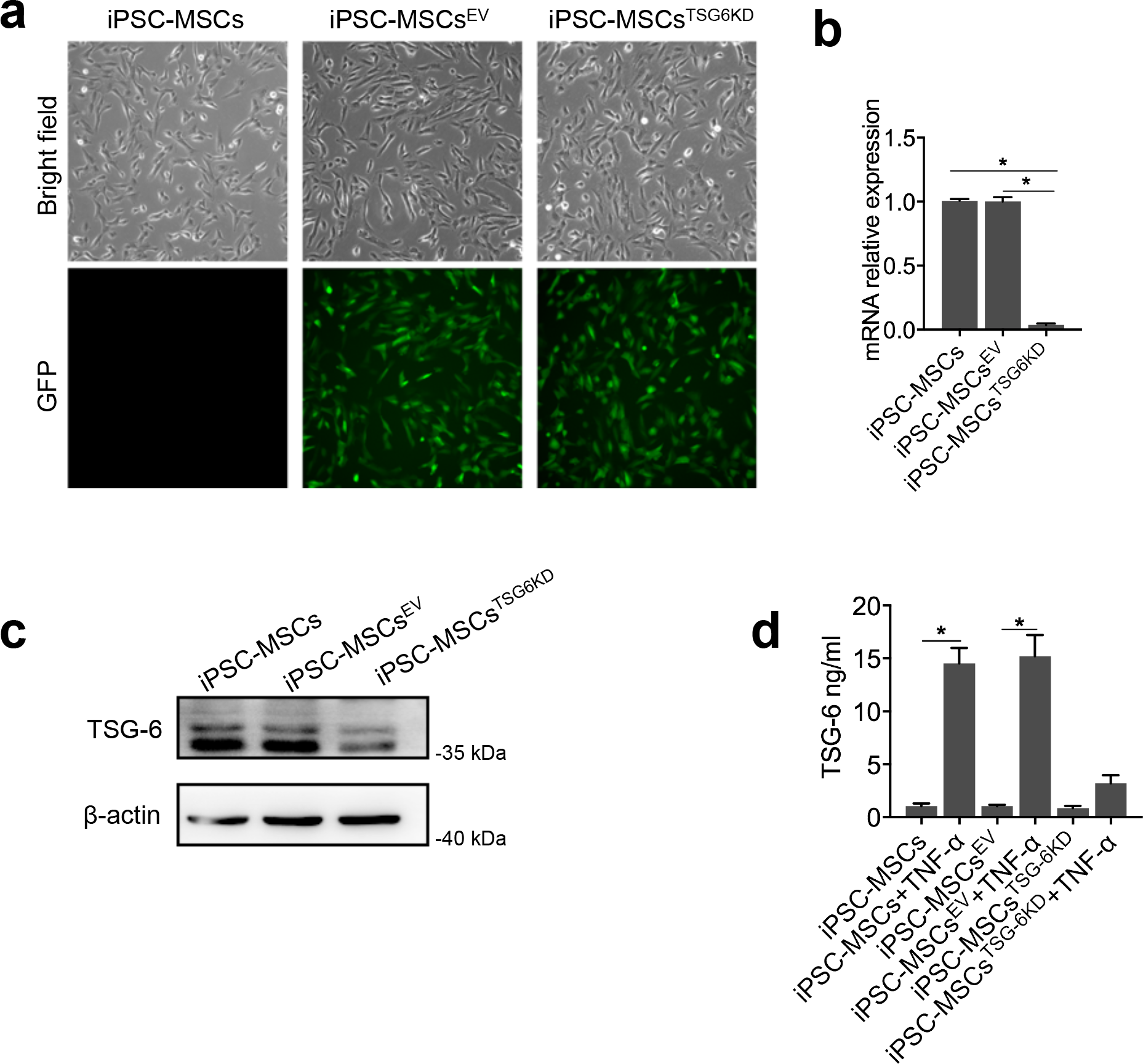

Supplement: Supplementary file 3 — Supplementary figure 2 [file 41419_2019_1957_MOESM3_ESM.tif]

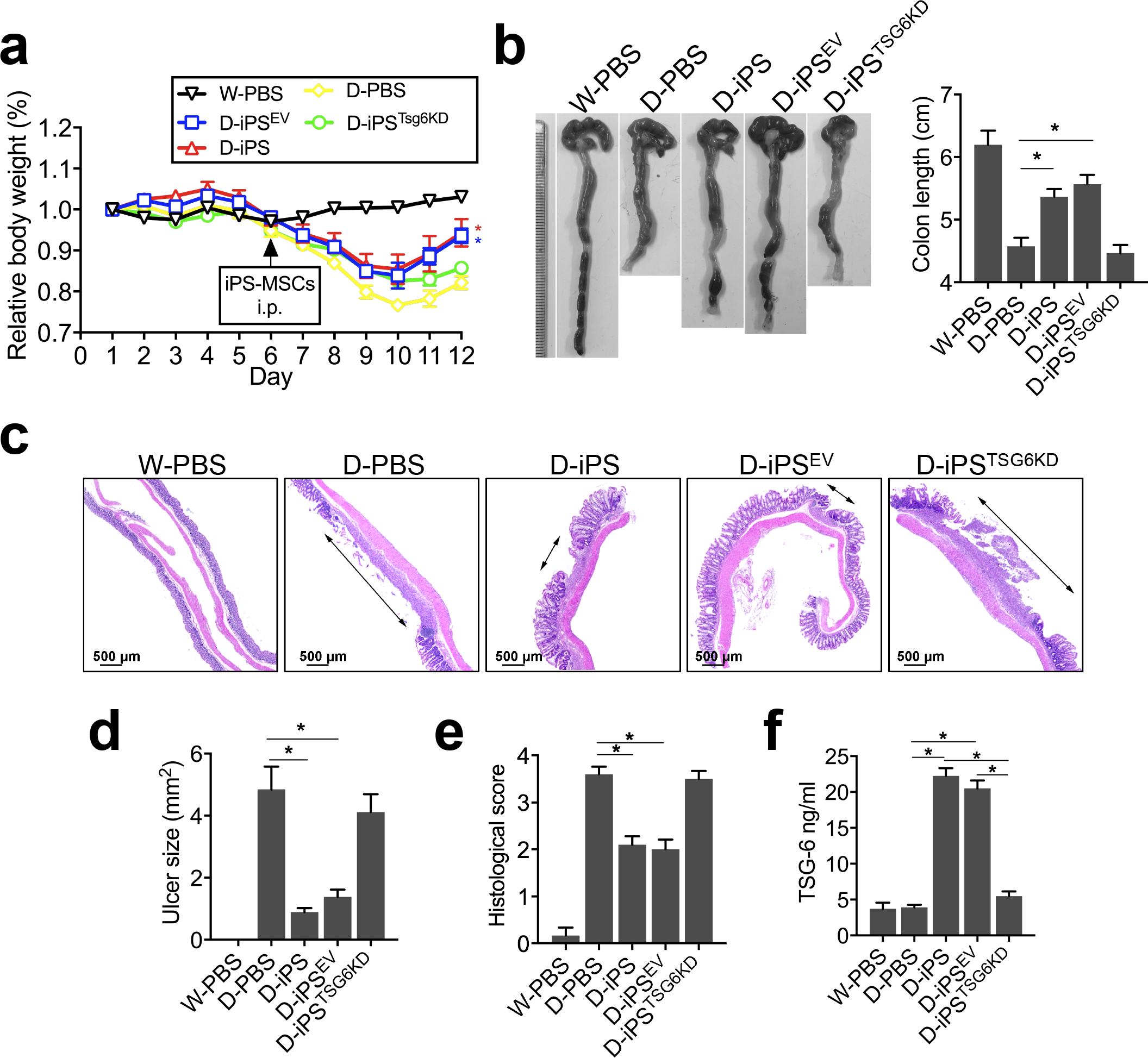

Supplement: Supplementary file 4 — Supplementary figure 3 [file 41419_2019_1957_MOESM4_ESM.tif]

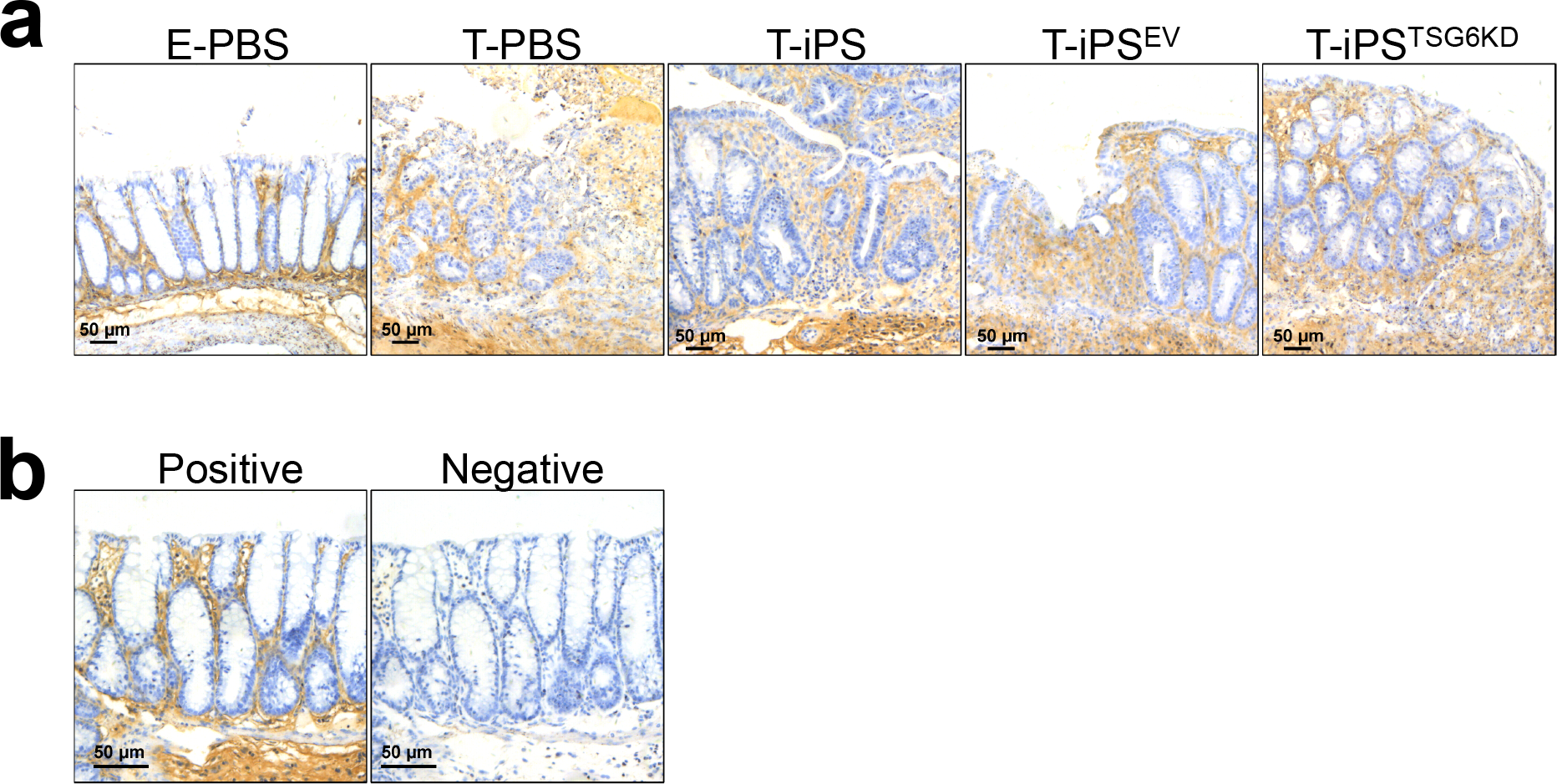

Supplement: Supplementary file 5 — Supplementary figure 4 [file 41419_2019_1957_MOESM5_ESM.tif]

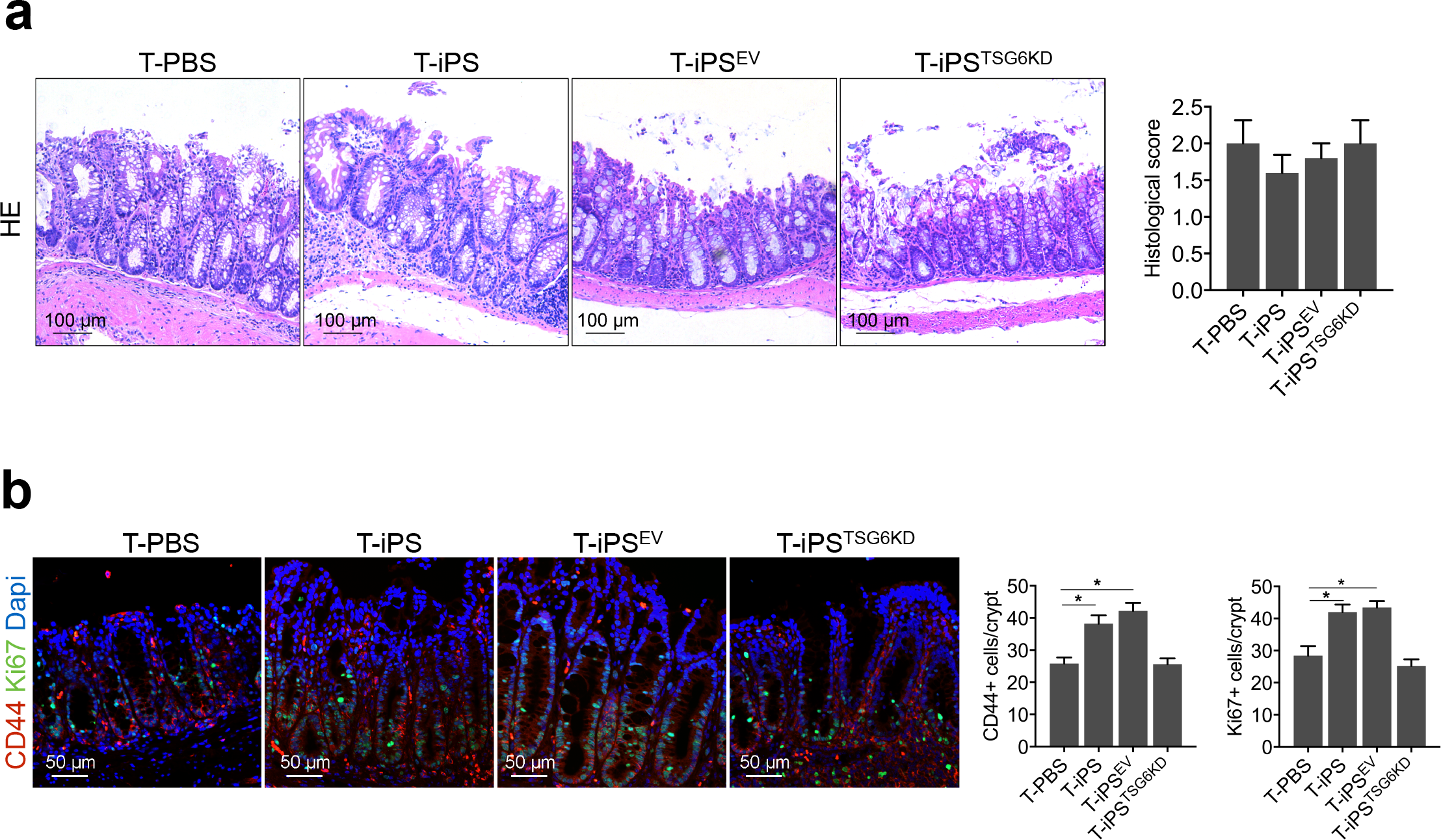

Supplement: Supplementary file 6 — Supplementary figure 5 [file 41419_2019_1957_MOESM6_ESM.tif]

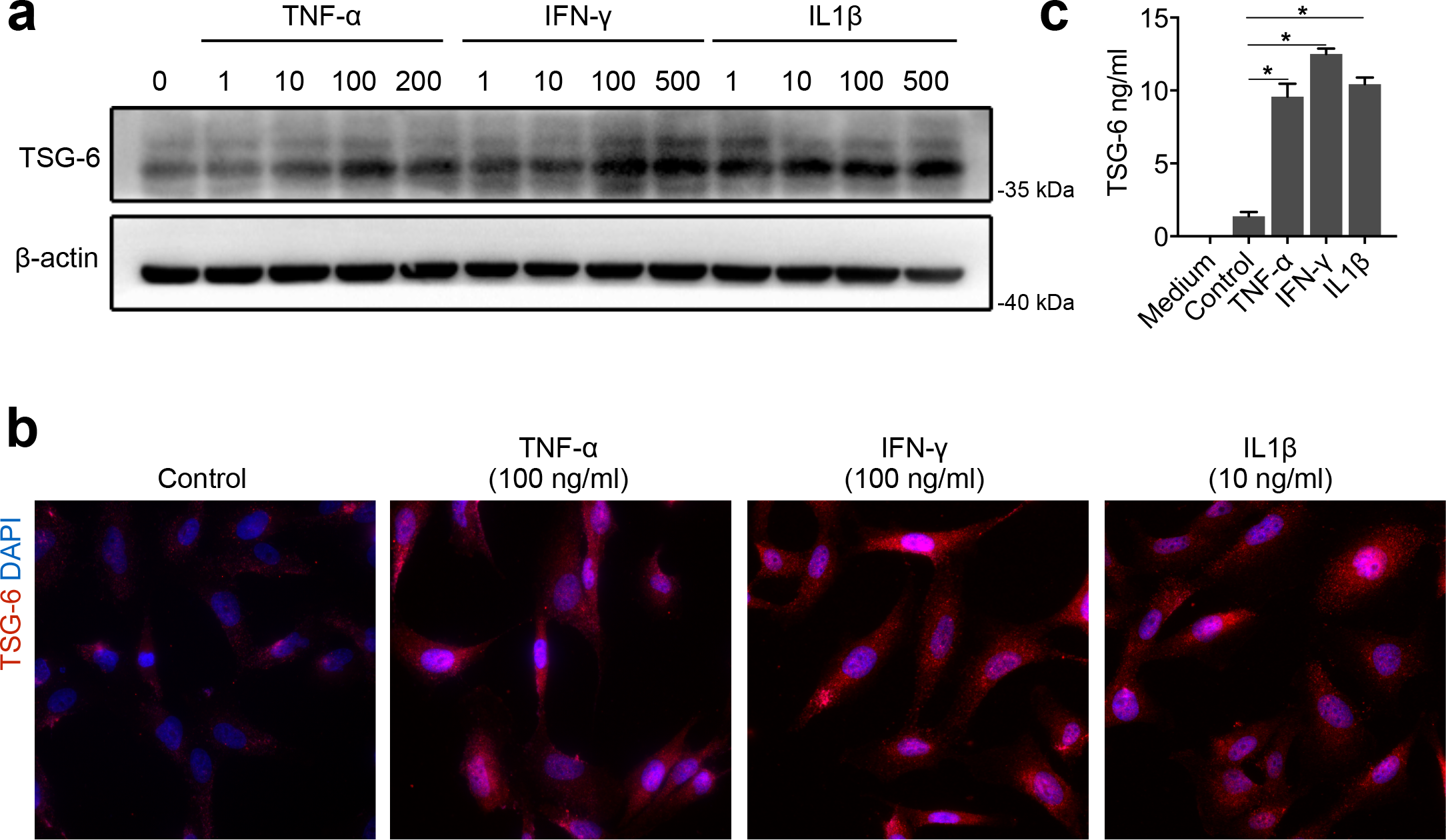

Supplement: Supplementary file 7 — Supplementary figure 6 [file 41419_2019_1957_MOESM7_ESM.tif]

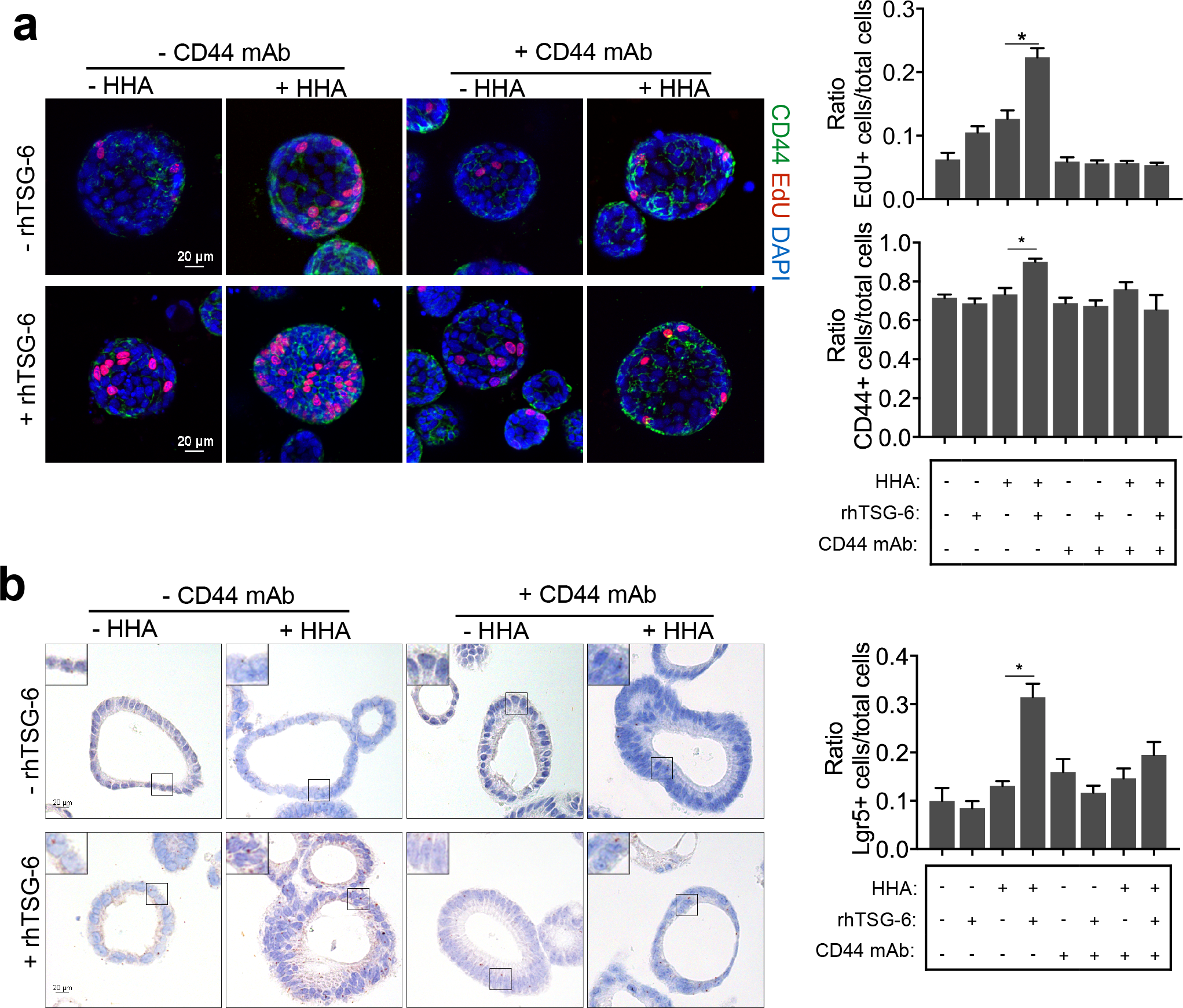

Supplement: Supplementary file 8 — Supplementary figure 7 [file 41419_2019_1957_MOESM8_ESM.tif]

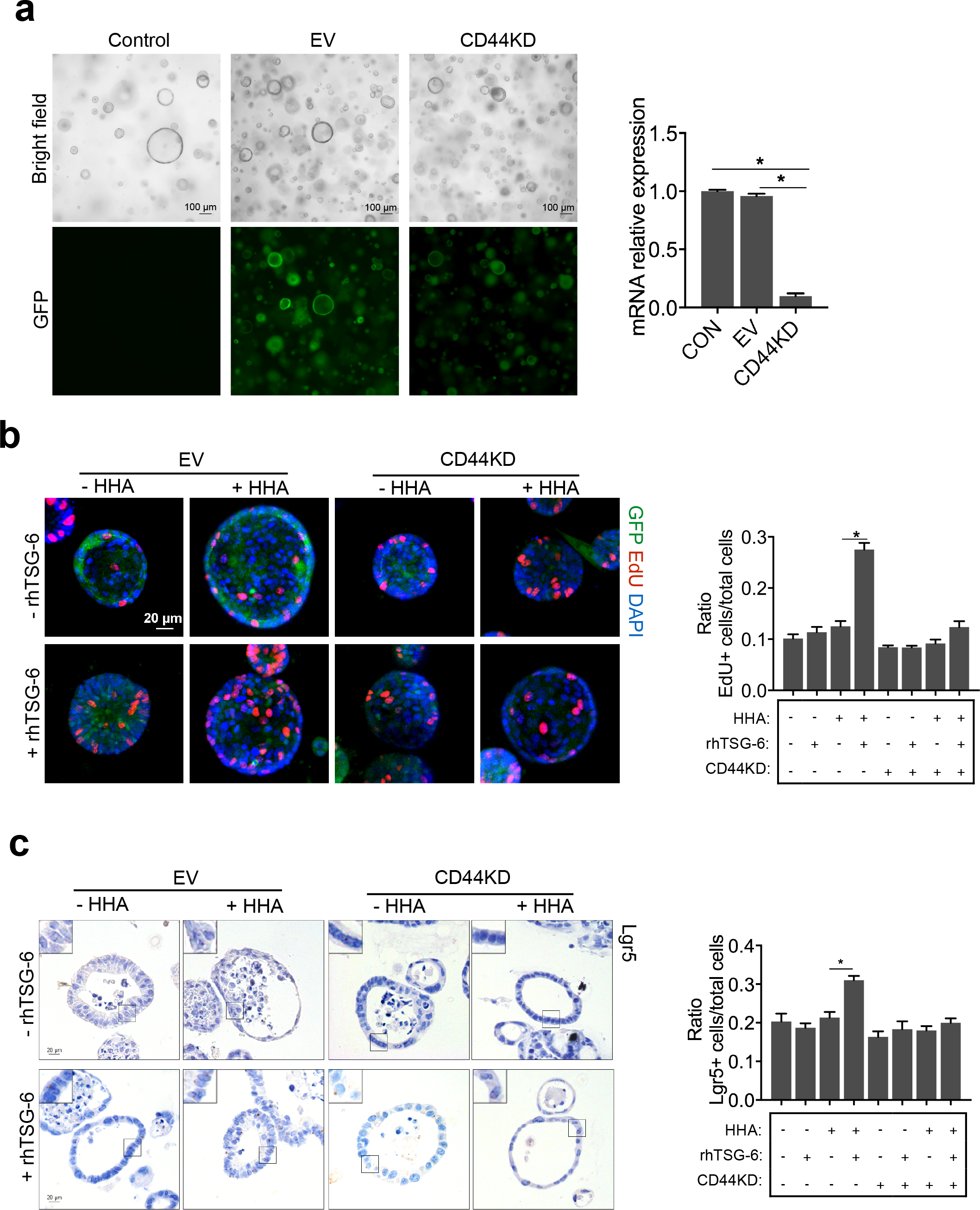

Supplement: Supplementary file 9 — Supplementary figure 8 [file 41419_2019_1957_MOESM9_ESM.tif]

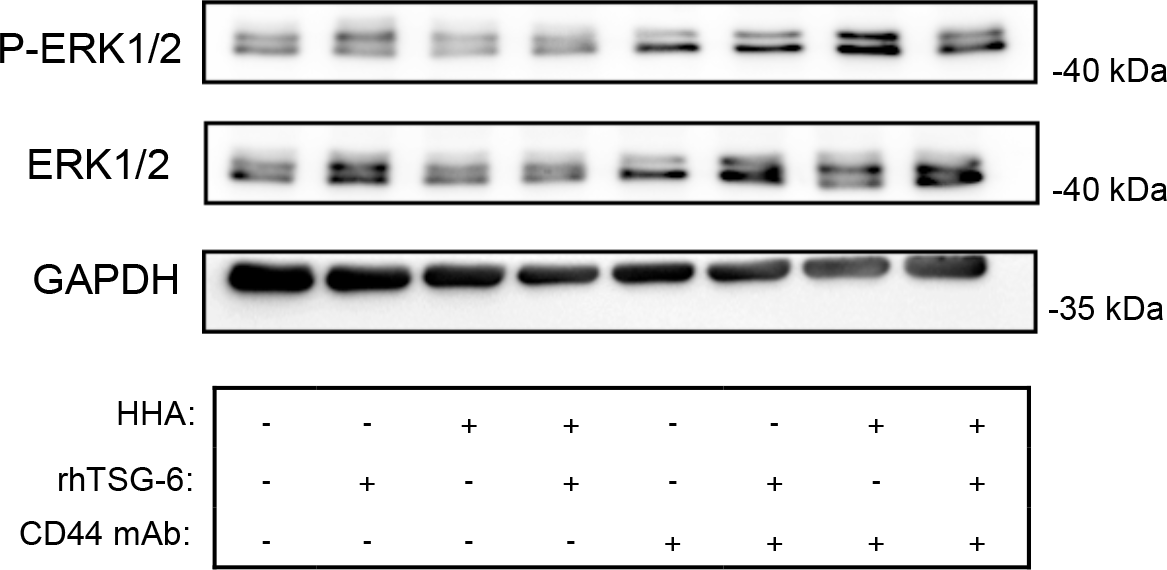

Supplement: Supplementary file 10 — Supplementary figure 9 [file 41419_2019_1957_MOESM10_ESM.tif]
